# Association of the polymorphisms of the cholesteryl ester transfer protein gene with coronary artery disease: a meta-analysis

**DOI:** 10.3389/fcvm.2023.1260679

**Published:** 2023-12-07

**Authors:** Ruizhe Zhang, Qingya Xie, Pingxi Xiao

**Affiliations:** ^1^ Department of Cardiology, Sir Run Run, Hospital, Nanjing Medical University, Nanjing, China; ^2^ Department of Cardiology, The Forth Affiliated Hospital, Nanjing Medical University, Nanjing, China

**Keywords:** coronary artery disease, single nucleotide polymorphisms, cholesteryl ester transfer protein, meta-analysis, review

## Abstract

**Aims:**

This meta-analysis aimed to assess the association of the polymorphisms of cholesterol ester transfer protein (*CETP*) rs708272 (G>A), rs5882 (G>A), rs1800775 (C>A), rs4783961 (G>A), rs247616 (C>T), rs5883 (C>T), rs1800776 (C>A), and rs1532624 (C>A) with coronary artery disease (CAD) and the related underlying mechanisms.

**Methods:**

A comprehensive search was performed using five databases such as PubMed, EMBASE, Web of Science, Cochrane Library and Scopus to obtain the appropriate articles. The quality of the included studies was assessed by the Newcastle-Ottawa Scale. The statistical analysis of the data was performed using STATA 17.0 software. The association between *CETP* gene polymorphisms and risk of CAD was estimated using the pooled odds ratio (OR) and 95% confidence interval (95% CI). The association of *CETP* gene polymorphisms with lipids and with CETP levels was assessed using the pooled standardized mean difference and corresponding 95% CI. *P* < 0.05 was considered statistically significant.

**Results:**

A total of 70 case-control studies with 30,619 cases and 31,836 controls from 46 articles were included. The results showed the *CETP* rs708272 polymorphism was significantly associated with a reduced risk of CAD under the allele model (OR* *=* *0.846, *P *< 0.001), the dominant model (OR* *=* *0.838, *P* < 0.001) and the recessive model (OR* *=* *0.758, *P *< 0.001). AA genotype and GA genotype corresponded to higher high-density lipoprotein cholesterol (HDL-C) concentrations in the blood compared with GG genotype across the studied groups (all *P* < 0.05). The *CETP* rs5882 and rs1800775 polymorphisms were not significantly associated with CAD under the allele model (*P* = 0.802, *P* = 0.392), the dominant model (*P* = 0.556, *P* = 0.183) and the recessive model (*P* = 0.429, *P* = 0.551). Similarly, the other mentioned gene polymorphisms were not significantly associated with CAD under the three genetic models.

**Conclusions:**

The *CETP* rs708272 polymorphism shows a significant association with CAD, and the carriers of the allele A are associated with a lower risk of CAD and higher HDL-C concentrations in the blood compared to the non-carriers. The *CETP* rs5882, rs1800775, rs4783961, rs247616, rs5883, rs1800776, and rs1532624 are not significantly associated with CAD.

**Systematic Review Registration:**

https://www.crd.york.ac.uk/prospero/display_record.php?ID=CRD42023432865, identifier: CRD42023432865.

## Introduction

**Figure 1:**
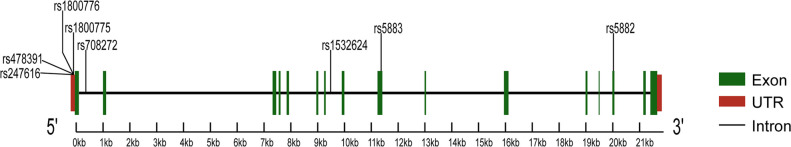
The structure of the *CETP* gene and the location of the mutations.

Characterized by the narrowing of coronary arteries, coronary artery disease (CAD) is the leading cause of morbidity and mortality worldwide (1). The etiology and progression of CAD involve complex interactions between genetic and environmental factors (2), the latter includes age, gender, hypertension, smoking, and dyslipidemia (3). Dyslipidemia refers to lipid abnormalities that include high levels of low-density lipoprotein cholesterol (LDL-C), triglycerides (TG) and total cholesterol (TC), as well as low levels of high-density lipoprotein cholesterol (HDL-C). Substantial advances in understanding the genetic basis of dyslipidemia have recently suggested that the cholesteryl ester transfer protein (CETP) is involved in the pathogenesis of CAD (4). CETP is a plasma glycoprotein that facilitates the exchange of triglycerides for cholesterol ester from HDL to apolipoprotein B-containing lipoproteins, reducing the concentration of HDL-C (5). CETP is encoded by the homonymous gene on chromosome 16q13, and its polymorphisms influence protein activity and plasma lipid profiles, thus affecting CAD development and progression (6). Many studies evaluated the association between the polymorphisms of the *CETP* gene and the risk of CAD to find the underlying mechanisms and potential clinical implications. Most of the studies focused on three polymorphisms in the *CETP* gene, such as rs708272, rs5882, and rs180075. The first (also known as TaqIB) is one of the most common polymorphisms, consisting of a G-to-A substitution in the 279th nucleotide in the first intron of the gene (7). The second polymorphism is characterized by a single nucleotide polymorphism (SNP) that leads to the substitution of an isoleucine (I) with a valine (V) at the position 405 of the CETP protein sequence (8). The third polymorphism is characterized by an SNP at position 629 of the *CETP* gene, which results in the substitution of C-to-A (9). Although a review already in 2008 reported that *CETP* rs708272, rs5882, and rs180075 polymorphisms are significantly associated with the CAD risk (10), some recent studies published contradictory results. This meta-analysis aims to assess the association between CAD and common CETP gene polymorphisms represented by three SNPs (rs708272, rs5882, rs1800775), as well as five uncommon SNPs (rs4783961, rs247616, rs5883, rs1800776, rs1532624). Figure 1 illustrates the CETP gene structure and mutation locations.

## Methods

This meta-analysis was performed in accordance with the guidelines outlined by the statement of the Preferred Reporting Items for Systematic Reviews and Meta-analyses (PRISMA) (11).

### Search strategy

We conducted a comprehensive search using the following databases: PubMed, EMBASE, Web of Science, Cochrane Library, and Scopus. The search spanned from January 1, 1988, to May 9, 2023. We used a comprehensive search strategy with the following keywords: “Cholesterol Ester Transfer Protein” or “CETP” and “Myocardial Infarction” or “Cardiovascular Stroke” or “Heart Attack” or “Coronary Disease” or “CAD” or “CHD” and “Polymorphism, Single Nucleotide” or “SNP” or “Genotype” or “mutant” or “variant”.

### Inclusion and exclusion criteria

Clinical case-control studies that assessed the associations between CETP gene polymorphisms and CAD were included if they provided sufficient information on genotype counts for CAD patients and controls, allowing us to calculate the odds ratio (OR) and 95% confidence interval (95% CI). Additionally, all cases had to meet the diagnostic criteria for CAD.

We excluded duplicates, as well as articles related to animal experiments, reviews, meta-analyses, case reports, meeting abstracts, letters, and editorial comments. Non-English-language publications and those with insufficient statistical results were also excluded.

### Data extraction and quality assessment

Two investigators, Ruizhe Zhang and Qingya Xie, independently extracted data from eligible articles using a standardized procedure that adhered to the inclusion and exclusion criteria. Any disagreements were resolved through discussion to achieve consensus. Data extracted from the articles included first authors' names, publication years, study population countries and ethnicities, diagnostic criteria (coronary stenosis or myocardial infarction), genotyping methods, case/control sources, age distributions of cases/controls, gender distributions of cases/controls, sample sizes of cases/controls, genotype counts in cases/controls, and *p*-values for Hardy-Weinberg Equilibrium (HWE). Additionally, if available, data on the associations of CETP gene polymorphisms with serum lipid concentrations and CETP levels, as well as mean and standard deviation values for CETP level, HDL-C, LDL-C, TG, and TC concentrations across genotypes, were extracted to elucidate underlying mechanisms.

The Newcastle-Ottawa Scale (NOS) was used to assess the quality of included studies. The NOS evaluation covered three aspects: selection (0–4), comparability (0–2), and outcome (0–3), with a total possible score ranging from 0 to 9 (12).

### Statistical analysis

Statistical analysis was performed using the STATA software (Version 17.0, Stata Corporation, College Station, TX, USA). We estimated the association between CETP gene polymorphisms and CAD risk using the pooled OR and its 95% CI under three genetic models: the allele model (M vs. W), the dominant model (MM + MW vs. WW), and the recessive model (MM vs. WW + MW), where W represents the wild allele, M represents the mutant allele, WW stands for wild homozygote, WM for heterozygote, and MM for mutant homozygote. We assessed the association of CETP gene polymorphisms with lipid serum concentrations and CETP levels using the pooled standardized mean difference (SMD) and its corresponding 95% CI under two models: the homozygote model (MM vs. WW) and the heterozygote model (MW vs. WW). The statistical significance of the pooled OR and pooled SMD was evaluated using the *Z* test. The significant threshold *P* < 0.05 was selected. Cochran's *Q*-statistic and *I^2^* tests were used to assess potential heterogeneity among the included studies. If the *Q*-test resulted in a *P *< 0.05 or *I^2 ^*> 50% indicating a significant heterogeneity, we employed a random-effects model. Otherwise, a fixed-effects model was used (13). The genotype count in the control was measured for HWE using a chi-square test. Sensitivity analysis was performed to assess the impact of individual studies on the overall pooled OR and SMD by sequentially excluding one study at a time and examining the effect. We conducted sensitivity analysis by systematically excluding one study at a time and examining its impact on the overall pooled OR and SMD. To evaluate publication bias, we used funnel plots, Begg's test, and Egger's test (14). A significance threshold of *P* < 0.05 was selected for determining the statistical significance of Begg's test and Egger's test. Additionally, we applied the trim-and-fill method to estimate the potential number of missing studies and their outcomes, which could contribute to publication bias.

## Results

### Flowchart and initial selection

**Figure 2:**
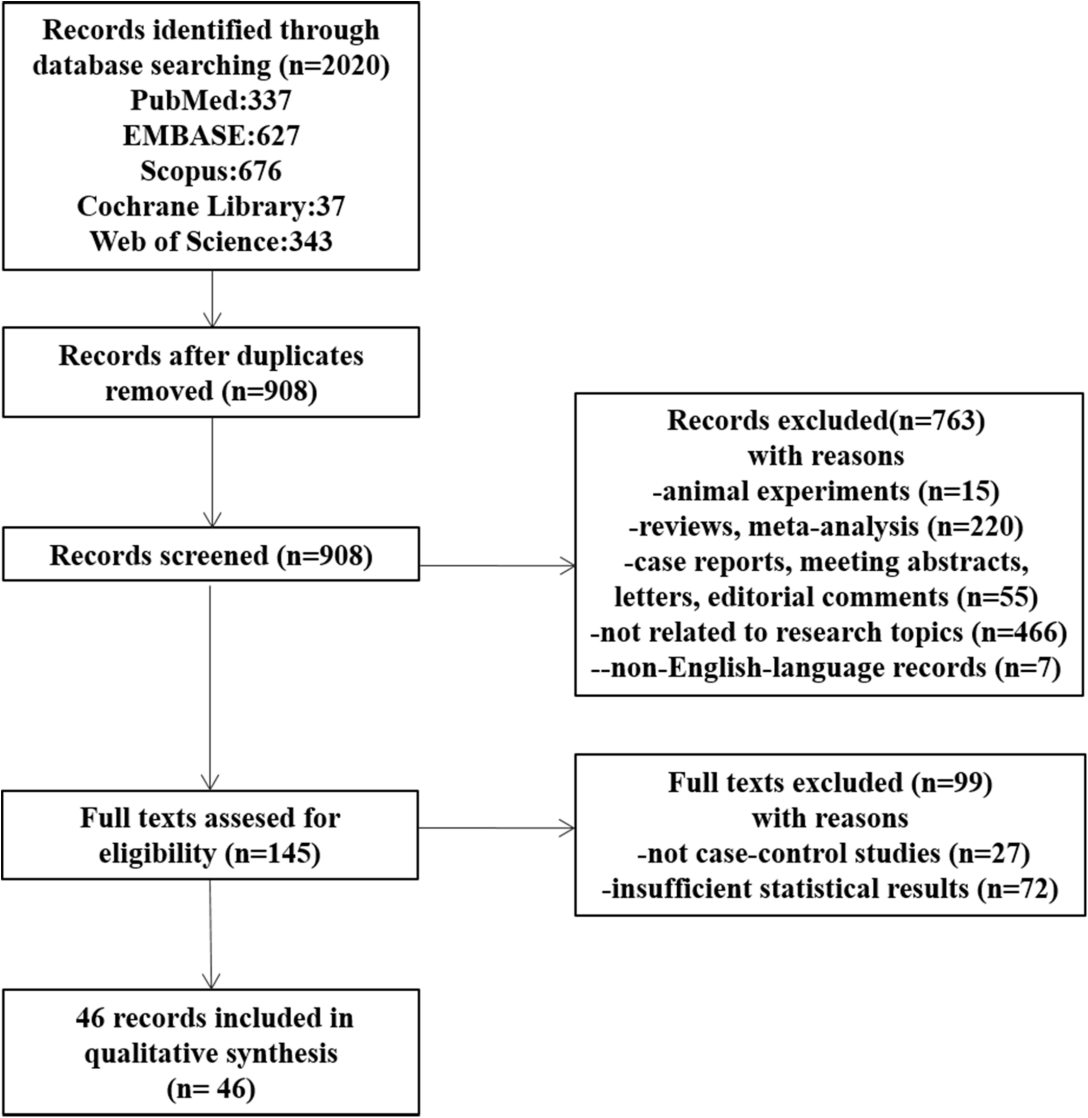
The flowchart shows the procedure for selecting studies.

 Figure 2 shows the flowchart of the details of the selection procedure. Initially, 908 articles were collected from five databases. A total of 763 publications were excluded based on their titles and abstracts, the full texts were evaluated, and additional 99 articles were removed. Finally, 46 articles meeting the inclusion criteria were included (15–60).

### Characteristics of included studies

Seventy case-control studies, meeting the inclusion criteria, were included in this meta-analysis, comprising a total of 30,619 cases and 31,836 controls from 46 articles. The main characteristics of the selected studies and the genotypic distributions of *CETP* gene polymorphisms are shown in Tables 1, 2.

**Table 1:** Main characteristics of the selected studies of CETP gene polymorphisms.

First author	Year	Country	Ethnicity	CAD subtype	Method	Source		Size		Age		Sex, M/F		SNPs	HWE test (*P* value)	NOS score
						Case	Control	Case	Control	Case	Control	Case	Control			
Rayat et al. (15)	2022	Iran	Caucasian	Coronary stenosis	PCR-RELP	HB	HB	100	100	57.61 ± 11.44	57.24 ± 11.20	37/64	64/47	rs708272(G>A)	0.2293	8
								100	100	57.61 ± 11.44	57.24 ± 11.20	37/64	64/47	rs5882(G>A)	0.6405	
Vargas et al. (16)	2021	Mexico	Caucasian	Coronary stenosis	NON-RFLP	HB	HB	216	604	59 ± 8.89	53 ± 8.15	170/49	NA	rs708272(G>A)	0.2342	6
								218	604	59 ± 8.89	53 ± 8.15	170/49	NA	rs4783961(G>A)	0.5007	
Raina et al. (17)	2020	India	Asian	Coronary stenosis	PCR-RELP	HB	HB	400	400	NA	NA	NA	NA	rs708272(G>A)	0.1444	7
Bordoni et al. (48)	2020	Poland	Caucasian	Coronary stenosis	NON-RFLP	HB	PB	394	153	66.4 ± 11.7	66.3 ± 8.1	269/125	269/125	rs247616(C>T)	0.3305	8
Amer et al. (18)	2019	Egypt	Caucasian	Myocardial infarction	NON-RFLP	HB	HB	36	26	55.83 ± 1.88	40.08 ± 1.05	29/7	16/10	rs708272(G>A)	0.8448	8
Arikan et al. (49)	2019	Turkey	Caucasian	Coronary stenosis	NON-RFLP	HB	HB	45	45	59.01 ± 10.72	58.02 ± 8.75	33/12	28/17	rs5883(C>T)	0.755	5
Mirhafez et al. (50)	2019	Iran	Caucasian	Coronary stenosis	NON-RFLP	HB	PB	190	95	NA	50.1 ± 10.5	107/87	31/65	rs5882(G>A)	0.6799	6
Iwanicka et al. (19)	2018	Poland	Caucasian	Coronary stenosis	NON-RFLP	HB	PB	239	240	44.62 ± 5.95	44.62 ± 5.95	173/75	173/75	rs708272(G>A)	0.1322	7
								235	213	44.62 ± 5.95	44.62 ± 5.95	173/75	173/75	rs247616(C>T)	0.1071	
								237	224	44.62 ± 5.95	44.62 ± 5.95	173/75	173/75	rs1532624(C>A)	** 0**.0308*	
Cai et al. (20)	2018	China	Asian	Coronary stenosis	NON-RFLP	HB	HB	557	414	64.24 ± 9.94	61.49 ± 9.11	391/165	214/200	rs708272(G>A)	0.1194	7
Wu et al. (51)	2018	China	Asian	Coronary stenosis	NON-RFLP	HB	PB	777	731	NA	NA	NA	NA	rs4783961(G>A)	0.3372	7
Maksoud et al. (21)	2017	Egypt	Caucasian	Myocardial infarction	NON-RFLP	HB	PB	40	30	55.48 ± 11.6	39.53 ± 5.26	32/8	20/10	rs708272(G>A)	0.6377	5
Devi et al. (52)	2017	India	Asian	Coronary stenosis	PCR-RELP	HB	HB	50	50	51.22 ± 7.60	48.0 ± 7.2	35/15	33/17	rs1800775(C>A)	0.4595	7
Cyrus et al. (22)	2016	Saudi Arabia	Caucasian	Coronary stenosis	NON-RFLP	HB	PB	990	618	58.37 ± 12.91	54.8 ± 8.5	708/282	423/195	rs708272(G>A)	0.1966	7
								879	618	58.37 ± 12.91	54.8 ± 8.5	708/282	423/195	rs5882(G>A)	0.3577	
Goodarzynejad et al. (53)	2016	Iran	Caucasian	Coronary stenosis	NON-RFLP	HB	HB	531	553	45.6 ± 5.8	45.5 ± 6.1	250/281	248/305	rs5882(G>A)	0.39	8
Ganesan et al. (54)	2016	India	Asian	Coronary stenosis	NON-RFLP	HB	HB	323	300	56.21 ± 10.45	65.26 ± 10.30	226/97	226/97	rs5883(C>T)	0.7013	6
Kaman et al. (23)	2015	Turkey	Caucasian	Coronary stenosis	PCR-RELP	HB	HB	210	100	NA	58.11 ± 7.68	67/173	47/53	rs708272(G>A)	0.3212	7
Mehlig et al. (24)	2014	Sweden	Caucasian	Coronary stenosis	NON-RFLP	PB	PB	618	2,921	NA	NA	435/165	1,378/453	rs708272(G>A)	0.5365	6
Abd El-Aziz et al. (25)	2014	Egypt	Caucasian	Coronary stenosis	PCR-RELP	HB	PB	116	119	42.4 ± 67.3	41.9 ± 66.4	90/26	63/56	rs708272(G>A)	0.6488	7
Zende et al. (55)	2014	India	Asian	Myocardial infarction	NON-RFLP	HB	PB	100	100	NA	NA	NA	NA	rs5882(G>A)	0.4047	6
Wang et al. (26)	2013	China	Asian	Coronary stenosis	NON-RFLP	HB	HB	418	420	66 ± 2.25	66 ± 2.5	167/253	168/256	rs708272(G>A)	0.8392	8
								418	421	66 ± 2.25	66 ± 2.5	167/253	168/256	rs1800775(C>A)	0.2096	
								421	424	66 ± 2.25	66 ± 2.5	167/253	168/256	rs5882(G>A)	0.1485	
								424	424	66 ± 2.25	66 ± 2.5	167/253	168/256	rs1532624(C>A)	0.2037	
Lu et al. (27)	2013	Singapore	Asian	Coronary stenosis	PCR-RELP	HB	PB	659	927	NA	NA	NA	NA	rs708272(G>A)	0.0553	7
								659	912	NA	NA	NA	NA	rs1800775(C>A)	0.3895	
Xu et al. (56)	2013	China	Asian	Coronary stenosis	NON-RFLP	HB	HB	289	330	62.07 ± 9.50	63.41 ± 9.21	210/80	245/86	rs247616(C>T)	0.0844	8
Rahimi et al. (28)	2011	Iran	Caucasian	Coronary stenosis	PCR-RELP	HB	HB	207	92	NA	54.3 ± 8.5	NA	47/45	rs708272(G>A)	0.2109	6
Kolovou et al. (29)	2011	Greece	Caucasian	Coronary stenosis	PCR-RELP	HB	HB	374	96	NA	NA	315/41	NA	rs708272(G>A)	0.5732	7
								374	96	NA	NA	315/41	NA	rs5882(G>A)	** 0**.0107*	
Corella et al. (30)	2010	Spanish	Caucasian	Coronary stenosis	PCR-RELP	PB	PB	557	1,180	53.9 ± 7.3	53.8 ± 7.2	445/112	932/428	rs708272(G>A)	0.5571	8
Poduri et al. (31)	2009	India	Asian	Coronary stenosis	NON-RFLP	HB	PB	265	150	47.52 ± 7.7	47.01 ± 8.1	222/43	114/36	rs708272(G>A)	0.252	7
								265	150	47.52 ± 7.7	47.01 ± 8.1	222/43	114/36	rs1800775(C>A)	0.1554	
								265	150	47.52 ± 7.7	47.01 ± 8.1	222/43	114/36	rs5882(G>A)	0.0958	
Kaestner et al. (33)	2009	Greece	Caucasian	Coronary stenosis	PCR-RELP	HB	HB	204	35	NA	NA	178/26	NA	rs708272(G>A)	0.7782	6
Padmaja et al. (32)	2009	India	Asian	Coronary stenosis	PCR-RELP	HB	HB	504	338	50.71 ± 8.64	49.66 ± 6.61	458/46	300/38	rs708272(G>A)	0.3862	8
								504	338	50.71 ± 8.64	49.66 ± 6.61	458/46	300/38	rs1800775(C>A)	** 0**.0079*	
								504	338	50.71 ± 8.64	49.66 ± 6.61	458/46	300/38	rs5882(G>A)	0.1027	
Tanrikulu et al (57)	2009	Turkey	Caucasian	Coronary stenosis	NON-RFLP	HB	HB	120	120	54 ± 9	52 ± 10	94/26	58/62	rs1800775(C>A)	0.9034	6
Rejeb et al. (34)	2008	Tunisia	Caucasian	Coronary stenosis	PCR-RELP	HB	HB	212	104	60.6 ± 10.6	59.4 ± 11.9	141/71	58/46	rs708272(G>A)	0.9590	6
Meiner et al. (35)	2008	USA	Caucasian	Myocardial infarction	NON-RFLP	PB	PB	550	620	NA	NA	321/256	308/351	rs708272(G>A)	0.1981	6
								557	629	NA	NA	321/256	308/351	rs4783961(G>A)	0.1822	
								556	628	NA	NA	321/256	308/351	rs1800775(C>A)	0.5039	
								562	630	NA	NA	321/256	308/351	rs5882(G>A)	0.1981	
Hsieh et al. (36)	2007	China	Asian	Coronary stenosis	PCR-RELP	HB	HB	101	264	64.34 ± 10.5	58.15 ± 12.31	57/44	148/116	rs708272(G>A)	0.9198	7
Dedoussis et al. (37)	2007	Greece	Caucasian	Myocardial infarction	PCR-RELP	HB	HB	237	237	58 ± 13	57 ± 7	182/52	185/52	rs708272(G>A)	0.5295	6
Zee et al. (58)	2006	USA	Caucasian	Myocardial infarction	NON-RFLP	PB	PB	523	2,092	58.3 ± 0.4	58.4 ± 0.2	523/0	2,092/0	rs1800775(C>A)	0.2399	6
Zheng et al. (59)	2005	China	Asian	Coronary stenosis	NON-RFLP	HB	HB	203	209	55.4 ± 6.5	54.8 ± 8.7	137/66	141/68	rs1800775(C>A)	0.5853	7
								203	209	55.4 ± 6.5	54.8 ± 8.7	137/663	141/68	rs5882(G>A)	0.5634	
Whiting et al. (39)	2005	USA	Caucasian	Coronary stenosis	PCR-RELP	PB	PB	2,392	827	65 ± 11	59 ± 13	2,522/797	706/679	rs708272(G>A)	** 0**.0388*	6
Falchi et al. (40)	2005	France	Caucasian	Coronary stenosis	PCR-RELP	HB	PB	100	100	46 ± 0.03	37 ± 0.04	85/15	60/40	rs708272(G>A)	0.581	5
Yilmaz et al. (38)	2005	Turkey	Caucasian	Coronary stenosis	PCR-RELP	HB	PB	173	111	52.6 ± 10.6	52.5 ± 12.6	119/54	68/49	rs708272(G>A)	0.0926	7
Keavney et al. (41)	2004	UK	Caucasian	Myocardial infarction	PCR-RELP	HB	PB	4,442	3,273	50.56 ± 0.12	46.26 ± 0.14	3,052/1,633	1,537/1,923	rs708272(G>A)	** 0**.0054*	8
Andrikopoulos et al. (42)	2004	Greece	Caucasian	Myocardial infarction	NON-RFLP	PB	PB	1,625	735	NA	NA	NA	NA	rs708272(G>A)	0.1901	5
Tobin et al.	2004	UK	Caucasian	Myocardial infarction	PCR-RELP	HB	HB	547	505	61.9 ± 9.2	58.6 ± 10.7	372/175	313/192	rs1800775(C>A)	0.468	7
								547	505	61.9 ± 9.2	58.6 ± 10.7	372/175	313/192	rs5882(G>A)	0.4559	
								547	505	61.9 ± 9.2	58.6 ± 10.7	372/175	313/192	rs1800776(C>A)	0.209	
Isbir et al. (43)	2003	Turkey	Caucasian	Coronary stenosis	PCR-RELP	HB	PB	87	69	52.61 ± 10.69	52.57 ± 12.68	119/54	68/49	rs708272(G>A)	0.3717	7
Freeman et al. (44)	2003	UK	Caucasian	Coronary stenosis	PCR-RELP	PB	PB	499	1,105	56.9 ± 5.1	56.7 ± 5.2	NA	NA	rs708272(G>A)	0.7325	8
								498	1,107	56.9 ± 5.1	56.7 ± 5.2	NA	NA	rs1800775(C>A)	0.886	
								498	1,105	56.9 ± 5.1	56.7 ± 5.2	NA	NA	rs5882(G>A)	0.4372	
								498	1,107	56.9 ± 5.1	56.7 ± 5.2	NA	NA	rs1800776(C>A)	0.8695	
Liu et al. (45)	2002	China	Asian	Myocardial infarction	PCR-RELP	PB	PB	384	384	59.5 ± 8.5	59.5 ± 8.3	384/0	384/0	rs708272(G>A)	0.628	8
Wu et al. (46)	2001	China	Asian	Coronary stenosis	NON-RFLP	HB	PB	149	274	NA	NA	138/62	155/130	rs708272(G>A)	** 0**.0072*	7
								195	283	NA	NA	138/62	155/130	rs5882(G>A)	0.6433	
Arca et al. (47)	2001	Italy	Caucasian	Coronary stenosis	PCR-RELP	HB	PB	408	180	59.6 ± 9.6	59.8 ± 11.6	340/75	93/95	rs708272(G>A)	0.1125	7

UK: United Kingdom, PCR-RELP: polymerase chain reaction-restriction fragment length polymorphisms, HB: hospital-based, PB: population-based, SNPs: single nucleotide polymorphisms, HWE: Hardy-Weinberg Equilibrium; NOS, Newcastle-Ottawa Scale; NA, not available.

Bolded values indicate *P* < 0.05.

* *P* < 0.05 by HWE test.

**Table 2:** The genotypic distributions of CETP gene polymorphisms.

First author	Year	SNPs	Genotypic distribution of case					Genotypic distribution of control				
			MM	WM	WW	M	W	MM	WM	WW	M	W
Rayat et al. (15)	2022	rs708272(G>A)	18	55	27	91	109	21	43	36	85	115
		rs5882(G>A)	15	45	40	75	125	20	52	28	92	108
Vargas et al. (16)	2021	rs708272(G>A)	52	109	55	213	219	187	285	132	659	549
		rs4783961(G>A)	65	118	35	248	188	138	310	156	586	622
Raina et al. (17)	2020	rs708272(G>A)	81	215	104	377	423	72	212	116	356	444
Bordoni et al. (48)	2020	rs247616(C>T)	43	173	178	259	529	17	76	60	110	196
Amer et al. (18)	2019	rs708272(G>A)	3	24	9	30	42	4	13	9	21	31
Arikan et al. (49)	2019	rs5883(C>T)	0	5	40	5	85	0	4	41	4	86
Mirhafez et al. (50)	2019	rs5882(G>A)	25	89	76	139	241	11	40	44	62	128
Iwanicka et al. (19)	2018	rs708272(G>A)	36	123	80	195	283	46	131	63	223	257
		rs247616(C>T)	22	94	119	138	332	26	112	75	164	262
		rs1532624(C>A)	41	119	77	201	273	44	128	52	216	232
Cai et al. (20)	2018	rs708272(G>A)	106	256	195	468	646	80	186	148	346	482
Wu et al. (51)	2018	rs4783961(G>A)	29	281	467	339	1215	39	240	452	318	1,144
Maksoud et al. (21)	2017	rs708272(G>A)	4	24	12	32	48	5	16	9	26	34
Devi et al. (52)	2017	rs1800775(C>A)	1	13	36	15	85	1	17	32	19	81
Cyrus et al. (22)	2016	rs708272(G>A)	160	454	376	774	1,206	114	321	183	549	687
		rs5882(G>A)	178	523	178	879	879	147	297	174	591	645
Goodarzynejad et al. (53)	2016	rs5882(G>A)	74	234	223	382	680	77	246	230	400	706
Ganesan et al. (54)	2016	rs5883(C>T)	0	18	305	18	628	0	13	287	13	587
Kaman et al. (23)	2015	rs708272(G>A)	44	81	85	169	251	29	45	26	103	97
Mehlig et al. (24)	2014	rs708272(G>A)	96	313	209	505	731	563	1,420	938	2,546	3,296
Abd El-Aziz et al. (25)	2014	rs708272(G>A)	18	60	38	96	136	32	57	30	121	117
Zende et al. (55)	2014	rs5882(G>A)	22	46	32	90	110	18	44	38	80	120
Wang et al. (26)	2013	rs708272(G>A)	50	192	176	292	544	74	207	139	355	485
		rs1800775(C>A)	100	216	102	416	420	83	222	116	388	454
		rs5882(G>A)	71	215	135	357	485	63	219	142	345	503
		rs1532624(C>A)	29	183	209	241	601	34	191	199	259	589
Lu et al. (27)	2013	rs708272(G>A)	109	322	228	540	778	191	491	245	873	981
		rs1800775(C>A)	163	331	165	657	661	243	468	201	954	870
Xu et al. (56)	2013	rs247616(C>T)	11	74	204	96	482	10	71	249	91	569
Rahimi et al. (28)	2011	rs708272(G>A)	6	144	57	156	258	20	52	20	92	92
Kolovou et al. (29)	2011	rs708272(G>A)	46	202	126	294	454	29	45	22	103	89
		rs5882(G>A)	41	171	162	253	495	4	52	40	60	132
Corella et al. (30)	2010	rs708272(G>A)	86	247	224	419	695	161	537	482	859	1,501
Poduri et al. (31)	2009	rs708272(G>A)	41	107	117	189	341	35	82	33	152	148
		rs1800775(C>A)	28	110	127	166	364	7	38	105	52	248
		rs5882(G>A)	39	110	116	188	342	7	36	107	50	250
Kaestner et al. (33)	2009	rs708272(G>A)	37	114	53	188	220	6	16	13	28	42
Padmaja et al. (32)	2009	rs708272(G>A)	77	264	163	418	590	91	161	86	343	333
		rs1800775(C>A)	79	235	190	393	615	49	129	160	227	449
		rs5882(G>A)	124	233	147	481	527	92	154	92	338	338
Tanrikulu et al (57)	2009	rs1800775(C>A)	27	56	37	110	130	22	58	40	102	138
Rejeb et al. (34)	2008	rs708272(G>A)	15	93	104	123	301	12	47	45	71	137
Meiner et al. (35)	2008	rs708272(G>A)	95	282	173	472	628	134	320	166	588	652
		rs4783961(G>A)	148	256	153	552	562	182	297	150	661	597
		rs1800775(C>A)	120	301	135	541	571	133	321	174	587	669
		rs5882(G>A)	69	247	246	385	739	83	270	277	436	824
Hsieh et al. (36)	2007	rs708272(G>A)	35	47	19	117	85	130	111	23	371	157
Dedoussis et al. (37)	2007	rs708272(G>A)	33	121	83	187	287	39	120	78	198	276
Zee et al. (58)	2006	rs1800775(C>A)	128	266	129	522	524	548	1,019	525	2,115	2,069
Zheng et al. (59)	2005	rs1800775(C>A)	40	104	59	184	222	42	99	68	183	235
		rs5882(G>A)	31	109	63	171	235	43	99	67	185	233
Whiting et al. (39)	2005	rs708272(G>A)	401	1,200	791	2,002	2,782	170	377	280	717	937
Falchi et al. (40)	2005	rs708272(G>A)	13	57	30	83	117	18	52	30	88	112
Yilmaz et al. (38)	2005	rs708272(G>A)	35	72	66	142	204	26	46	39	98	124
Keavney et al. (41)	2004	rs708272(G>A)	790	2,175	1,477	3,755	5,129	646	1,527	1,100	2,819	3,727
Andrikopoulos et al. (42)	2004	rs708272(G>A)	190	741	694	1,121	2,129	87	355	293	529	941
Tobin et al.	2004	rs1800775(C>A)	111	293	143	515	579	140	244	121	524	486
		rs5882(G>A)	58	248	241	364	730	62	219	224	343	667
		rs1800776(C>A)	2	88	457	92	1,002	1	76	428	78	932
Isbir et al. (43)	2003	rs708272(G>A)	11	30	46	52	122	7	35	27	49	89
Freeman et al. (44)	2003	rs708272(G>A)	76	259	164	411	587	225	541	339	991	1,219
		rs1800775(C>A)	98	261	139	457	539	270	551	286	1,091	1,123
		rs5882(G>A)	49	211	238	309	687	225	541	339	991	1,219
		rs1800776(C>A)	7	71	420	85	911	6	146	955	158	2,056
Liu et al. (45)	2002	rs708272(G>A)	63	196	125	322	446	69	193	122	331	437
Wu et al. (46)	2001	rs708272(G>A)	25	79	45	129	169	52	159	63	263	285
		rs5882(G>A)	22	110	63	154	236	45	131	107	221	345
Arca et al. (47)	2001	rs708272(G>A)	68	187	153	323	493	36	77	67	149	211

SNPs, single nucleotide polymorphisms; W, wild allele; M, mutant allele; WW, wild homozygote; WM, heterozygote; MM, mutant homozygote.

Among these eligible studies, 33 involved the relationship between *CETP* rs708272 polymorphisms and CAD (15–47). Other 37 studied five other polymorphisms in the *CETP* gene, and 14 of them focused on rs5882 (15, 22, 26, 29, 31, 32, 35, 44, 46, 50, 53, 55, 59, 60), 11 on rs1800775 (26, 27, 31, 32, 35, 44, 52, 57–60)3 on rs4783961 (16, 35, 51), 3 on rs247616 (19, 48, 56), 2 on rs5883 (49, 54), 2 on rs1800776 (44, 60), and 2 on rs1532624 (19, 26). In addition, 6 studies deviated from HWE (19, 30, 32, 39, 41, 46). Among all 46 articles, 10 mentioned information on lipid serum concentrations including HDL-C, LDL-C, TG and TC for rs708272 (19–21, 23, 25, 26, 30, 31, 38, 44). One article was on the association between *CETP* rs708272 polymorphism and CETP level (23).

### Study populations and diagnostic criteria

Thirty-one articles mentioned studies performed among Caucasians (15, 16, 18, 19, 21–25, 28–30, 33–35, 37–44, 48–50, 53, 57, 58, 60), and 15 among Asians (17, 20, 26, 27, 31, 32, 36, 45, 46, 51, 52, 54–56, 59).

Thirty-six articles involved cases with coronary stenosis (15–17, 19, 20, 22–34, 36, 38–40, 43, 44, 46–54, 56, 57, 59), and 10 involved cases with myocardial infarction (18, 21, 35, 37, 41, 42, 45, 55, 58, 60).

### Genotyping methods

Genotyping methods varied among the included studies. Polymerase chain reaction-restriction fragment length polymorphisms (PCR-RFLP) is a molecular biology technology that analyzes genetic polymorphisms and sequence variations in DNA samples by combining the strength of PCR amplification with the precision of restriction enzyme cleavage. It offers relatively high resolution. NON-RFLP techniques employ methods that do not depend on restriction enzyme cleavage, such as TaqMan PCR, ARMS-PCR, MassARRAY and other approaches. Its resolution and specificity depend on the chosen approach. Twenty-three articles performed the genotyping using PCR-RELP (16, 18–22, 24, 26, 31, 35, 42, 46, 48–51, 53–59), and 23 used other methods (NON-RELP) (15, 17, 23, 25, 27–30, 32–34, 36–41, 43–45, 47, 52, 60).

### Control types and sample sizes

Control types and sample sizes varied across the studies.

Hospital-based (HB) control was used in 22 articles (15–18, 20, 23, 26, 28, 29, 32–34, 36, 37, 49, 52–54, 56, 57, 59, 60), and population-based (PB) control was used in 24 articles (19, 21, 22, 24, 25, 27, 30, 31, 35, 38–48, 50, 51, 55, 58).

Fourteen articles used a sample size of coronary cases ≥500 (20, 22, 24, 27, 30, 32, 35, 39, 41, 42, 51, 53, 58, 60), while 32 had used a sample size of coronary cases <500 (15–19, 21, 23, 25, 26, 28, 29, 31, 33, 34, 36–38, 40, 43–50, 52, 54–57, 59). Fourteen articles used a sample size of the controls ≥500 (16, 22, 24, 27, 30, 35, 39, 41, 42, 44, 51, 53, 58, 60), while 32 used a sample size of the controls <500 (15, 17–21, 23, 25, 26, 28, 29, 31–34, 36–38, 40, 43, 45–50, 52, 54–57, 59).

### Gender and age

Gender and age are known to be influential factors in the association between SNPs and the occurrence of CAD. Gender ratio and mean age varied across the studies.

There were 35 articles where man predominated in the case groups (16, 18–22, 24, 25, 29–41, 43, 45–50, 52, 54, 56–60), 4 articles where women did (15, 23, 26, 53), and 7 articles with insufficient information on the gender distribution in the case groups (17, 27, 28, 42, 44, 51, 55). There were 29 articles where men predominated in the control groups (18–22, 24, 25, 30–32, 34–40, 43, 45–49, 52, 54, 58–60), 8 articles where women did (23, 26, 35, 41, 47, 50, 53, 57), and 9 articles with insufficient information on the gender distribution in the control groups (16, 17, 27, 29, 33, 42, 44, 51, 55).

There were 21 articles with a mean age of the cases greater than or equal to 55 years (15, 16, 18, 20–22, 26, 34, 36, 37, 39, 44, 45, 47–49, 54, 56, 58–60), 12 articles with a mean age of cases less than 55 years (19, 25, 30–33, 38, 40, 43, 52, 53, 57), and 13 articles lacking sufficient information on the age distributions of cases (17, 23, 24, 27–29, 33, 35, 42, 46, 50, 51, 55). There were 17 articles with a mean age of controls greater than or equal to 55 years (15, 20, 23, 26, 34, 36, 37, 39, 44, 45, 47–49, 54, 56, 58, 60), 19 articles with a mean age of the controls less than 55 years (16, 18, 19, 21, 22, 25, 28, 30–32, 38, 40, 41, 43, 50, 52, 53, 57, 59), and 10 articles lacking sufficient information on the age distributions of controls (17, 24, 27, 29, 33, 35, 42, 46, 51, 55).

The NOS scores of the included articles were ≥5 and the average was 6.8.

### Association of the three common *CETP gene* polymorphisms with CAD

The details of the overall and subgroup analyses of the association of the *CETP* rs708272, rs5882 and rs180075 polymorphisms with CAD are listed in Table 3.

**Table 3:** Overall and subgroup analysis of the association of the *CETP* rs708272, rs5882 and rs180075 polymorphisms with CAD.

Subgroups	Studies (*n*)	Case/Control (*n*/*n*)	Allele model (M vs. W)					Dominant model (MM + WM vs. WW)					Recessive model (MM vs. WW + WM)				
			OR	95% CI		*P* value	*I* ^2^	OR	95% CI		*P* value	*I* ^2^	OR	95% CI		*P* value	*I* ^2^
				LL	UL				LL	UL				LL	UL		
rs708272 (G>A)																	
Overall	33	18069/17,093	0.846	0.798	0.897	**<0**.001*	59.5%	0.838	0.769	0.913	**<0**.001*	57.5%	0.758	0.687	0.836	**<0**.001*	51.4%
Based on HWE	30	11,086/12,719	0.836	0.785	0.891	**<0**.001*	56.5%	0.820	0.747	0.899	**<0**.001*	50.8%	0.739	0.657	0.830	**<0**.001*	53.4%
Based on ethnicity																	
Caucasian	24	14,632/13,522	0.873	0.825	0.925	**<0**.001*	41.8%	0.888	0.820	0.963	**0**.004*	35.6%	0.761	0.677	0.855	**<0**.001*	50.4%
Asian	9	3,437/3,571	0.945	0.898	0.995	** 0**.002*	75.9%	0.733	0.592	0.908	**0**.004*	73.7%	0.752	0.620	0.912	** 0**.004*	57.2%
Based on CAD subtype																	
Coronary stenosis	26	10,755/11,788	0.821	0.763	0.883	**<0**.001*	62.0%	0.806	0.724	0.898	**<0**.001*	60.2%	0.735	0.649	0.832	**<0**.001*	56.5%
Myocardial infarction	7	7,314/5,305	0.849	0.802	0.899	** 0**.032*	0.0%	0.965	0.895	1.041	0.361	0.0%	0.874	0.795	0.960	** 0**.005*	0.0%
Based on genotyping methods																	
NON-RFLP	12	5,703/7,052	0.835	0.771	0.904	**<0**.001*	43.4%	0.780	0.680	0.894	**<0**.001*	56.0%	0.803	0.725	0.890	**<0**.001*	0.0%
PCR-RFLP	21	12,366/10,041	0.853	0.787	0.924	**<0**.001*	64.4%	0.880	0.792	0.978	** 0**.017*	52.4%	0.727	0.627	0.843	**<0**.001*	65.4%
Based on source of control																	
HB	14	3,776/3,230	0.807	0.707	0.921	** 0**.001*	67.2%	0.835	0.703	0.992	** 0**.040*	53.8%	0.633	0.494	0.810	**<0**.001*	69.1%
PB	19	14,923/13,863	0.875	0.826	0.927	** 0**.023*	47.1%	0.842	0.763	0.930	** 0**.001*	60.8%	0.835	0.782	0.892	**<0**.001*	0.0%
Based on sample size of case																	
>=500	10	12,894/11,853	0.907	0.847	0.972	** 0**.006*	60.4%	0.883	0.795	0.980	** 0**.020*	66.0%	0.831	0.743	0.930	** 0**.001*	53.6%
<500	23	5,175/5,240	0.813	0.751	0.880	**<0**.001*	47.3%	0.799	0.699	0.914	** 0**.001*	49.6%	0.689	0.592	0.803	**<0**.001*	44.5%
Based on sample size of control																	
>=500	10	12,548/12,810	0.896	0.864	0.950	** 0**.001*	49.7%	0.884	0.801	0.976	** 0**.015*	61.8%	0.835	0.768	0.908	**<0**.001*	20.5%
<500	23	5,521/4,283	0.807	0.732	0.889	**<0**.001*	57.9%	0.799	0.695	0.918	**<0**.001*	52.8%	0.683	0.577	0.810	**<0**.001*	51.4%
Based on gender ratio of case																	
M/F >=1	25	13,951/13,214	0.848	0.773	0.907	**<0**.001*	57.5%	0.930	0.692	1.250	**0**.001*	56.2%	0.766	0.693	0.847	**<0**.001*	39.0%
M/F < 1	3	728/620	0.786	0.594	1.039	0.091	60.7%	0.784	0.467	1.319	0.360	73.8%	0.668	0.736	1.475	** 0**.006*	0.0%
NA	5	3,390/3,259	0.869	0.753	1.002	0.054	70.5%	1.070	0.793	1.444	0.096	54.5%	0.501	0.491	0.891	0.083	83.6%
Based on gender ratio of control																	
M/F >=1	21	8,064/8,598	0.838	0.976	0.907	**<0**.001*	54.2%	0.825	0.731	0.932	** 0**.002*	55.2%	0.739	0.640	0.854	**<0**.001*	51.8%
M/F < 1	5	6,028/4,593	0.838	0.725	0.968	** 0**.016*	70.3%	0.818	0.655	1.020	0.075	72.1%	0.834	0.755	0.921	**<0**.001*	0.0%
NA	7	3,977/3,902	0.867	0.757	0.991	** 0**.037*	69.0%	0.867	0.730	1.029	0.102	56.2%	0.769	0.602	0.983	0.036	68.8%
Based on mean age of case																	
>=55	14	6,590/5,313	0.870	0.812	0.933	**<0**.001*	26.4%	0.881	0.778	0.997	** 0**.045*	45.7%	0.773	0.699	0.854	**<0**.001*	0.0%
<55	10	6,483/5,580	0.812	0.701	0.939	** 0**.005*	74.7%	0.772	0.626	0.952	**0**.015*	72.6%	0.760	0.611	0.945	** 0**.014*	60.9%
NA	9	4,596/6,200	0.831	0.741	0.932	** 0**.002*	66.5%	0.819	0.708	0.949	** 0**.008*	52.3%	0.709	0.553	0.908	** 0**.007*	74.9%
Based on mean age of control																	
>=55	12	6,508/4,753	0.858	0.790	0.933	**<0**.001*	46.0%	0.853	0.739	0.984	** 0**.029*	58.7%	0.776	0.701	0.860	**<0**.001*	0.0%
<55	13	6,982/6,332	0.804	0.709	0.910	** 0**.001*	68.5%	0.797	0.671	0.947	** 0**.010*	61.6%	0.677	0.535	0.857	** 0**.001*	69.6%
NA	8	4,579/6,008	0.871	0.778	0.975	** 0**.016*	64.1%	0.849	0.731	0.987	** 0**.033*	54.2%	0.799	0.652	0.978	** 0**.030*	61.6%
rs5882 (G>A)																	
Overall	14	5,369/5,206	1.021	0.870	1.198	0.802	85.8%	1.071	0.853	1.344	0.556	85.7%	0.918	0.743	1.135	0.429	68.0%
Based on HWE	13	4,995/5,110	1.015	0.858	1.200	0.866	86.8%	1.081	0.850	1.375	0.524	86.8%	0.883	0.719	1.084	0.234	66.2%
Based on ethnicity																	
Caucasian	8	3,681/3,702	0.922	0.755	1.125	0.421	86.3%	0.930	0.692	1.250	0.632	87.7%	0.843	0.641	1.107	0.219	69.5%
Asian	6	1,688/1,504	1.181	0.902	1.548	0.226	84.3%	1.305	0.924	1.842	0.130	79.5%	1.042	0.736	1.475	0.818	65.8%
Based on CAD subtype																	
Coronary stenosis	11	4,160/3,971	1.020	0.829	1.254	0.852	88.9%	1.070	0.793	1.444	0.658	88.9%	0.920	0.702	1.205	0.543	74.3%
Myocardial infarction	3	1,209/1,235	0.997	0.886	1.122	0.966	0.0%	1.030	0.877	1.209	0.722	0.0%	0.928	0.731	1.178	0.537	0.0%
Based on genotyping method																	
NON-RFLP	9	3,346/3,062	1.138	0.976	1.327	0.098	74.5%	1.288	1.037	1.599	** 0**.022*	73.6%	0.989	0.798	1.225	0.917	50.9%
PCR-RFLP	5	2,023/2,144	0.823	0.621	1.090	0.174	87.0%	0.757	0.530	1.080	0.125	83.6%	0.799	0.513	1.244	0.321	78.2%
Based on source of control																	
HB	7	2,680/2,225	0.972	0.894	1.057	0.506	0.0%	0.973	0.864	1.097	0.655	0.0%	0.939	0.775	1.139	0.525	25.5%
PB	7	2,689/2,981	1.114	0.807	1.538	0.512	93.1%	1.246	0.788	1.972	0.347	93.1%	0.924	0.630	1.354	0.685	79.9%
Based on sample size of case																	
>=500	5	3,023/2,644	1.000	0.926	1.079	0.995	0.0%	1.077	0.895	1.295	0.717	63.3%	0.878	0.762	1.010	0.069	0.0%
<500	9	2,346/2,562	1.055	0.780	1.428	0.728	90.8%	1.076	0.722	1.604	0.431	89.4%	1.017	0.666	1.553	0.937	79.6%
Based on sample size of control																	
>=500	5	3,017/3,411	0.894	0.696	1.148	0.378	91.3%	0.942	0.644	1.378	0.758	92.4%	0.769	0.575	1.029	0.077	75.2%
<500	9	2,352/1,759	1.117	0.908	1.372	0.295	78.2%	1.168	0.889	1.536	0.265	72.4%	1.075	0.796	1.450	0.639	59.1%
Based on gender ratio of case																	
M/F >=1	8	3,516/2,715	1.134	0.954	1.348	0.155	78.7%	1.256	0.982	1.607	0.069	78.7%	0.994	0.773	1.279	0.965	59.3%
M/F < 1	4	1,255/1,286	0.970	0.852	1.130	0.645	19.4%	0.976	0.817	1.165	0.785	11.4%	0.951	0.756	1.196	0.668	8.9%
NA	2	598/1,205	0.806	0.370	1.758	0.588	92.5%	0.764	0.290	2.011	0.585	89.7%	0.708	0.241	2.079	0.530	87.3%
Based on gender ratio of control																	
M/F >=1	7	2,693/2,203	1.073	0.863	1.333	0.527	83.6%	1.216	0.888	1.666	0.223	83.0%	0.869	0.679	1.111	0.262	53.6%
M/F < 1	4	1,704/1,702	1.023	0.927	1.473	0.647	0.0%	1.033	0.900	1.186	0.646	0.0%	1.026	0.844	1.248	0.796	0.0%
NA	3	972/1,301	0.896	0.503	1.595	0.709	91.4%	0.803	0.430	1.502	0.493	86.0%	1.066	0.353	3.216	0.910	88.3%
Based on mean age of case																	
>=55	6	2,648/2,961	0.866	0.673	1.116	0.268	89.5%	0.900	0.604	1.343	0.607	91.0%	0.766	0.693	1.004	0.053	71.5%
<55	3	1,300/1,041	1.321	0.791	2.204	0.287	93.5%	1.388	0.720	2.675	0.327	92.2%	1.254	0.724	2.173	0.419	79.0%
NA	5	1,421/1,204	1.047	0.931	1.179	0.442	0.0%	1.091	0.927	1.270	0.294	0.0%	1.042	0.733	1.482	0.818	39.6%
Based on mean age of control																	
>=55	4	1,566/2,134	0.802	0.564	1.140	0.218	91.3%	0.753	0.481	1.180	0.215	89.3%	0.745	0.553	1.004	0.228	82.2%
<55	6	2,572/1,963	1.164	0.920	1.473	0.206	84.6%	1.328	0.954	1.848	0.093	83.3%	0.834	0.755	0.921	0.895	60.9%
NA	4	1,231/1,109	1.032	0.911	1.169	0.619	0.0%	1.069	0.900	1.029	0.449	0.0%	0.769	0.602	0.983	0.835	53.2%
rs1800775 (C>A)																	
Overall	11	4,343/6,532	1.055	0.934	1.191	0.392	73.9%	1.121	0.947	1.327	0.183	67.9%	0.957	0.826	1.107	0.551	45.8%
Based on HWE	10	3,839/6,194	1.033	0.911	1.170	0.617	87.9%	1.084	0.913	1.286	0.359	84.0%	0.946	0.808	1.108	0.494	76.8%
Based on ethnicity																	
Caucasian	5	2,244/4,452	0.949	0.853	1.055	0.334	45.9%	1.002	0.888	1.130	0.978	0.0%	0.859	0.717	1.028	0.098	46.2%
Asian	6	2,099/2,080	1.177	0.940	1.473	0.157	79.8%	1.245	0.901	1.721	0.184	79.4%	1.089	0.888	1.337	0.412	26.4%
Based on CAD subtype																	
Coronary stenosis	8	2,717/3,307	0.957	0.829	1.104	0.222	77.9%	1.173	0.914	1.506	0.211	75.2%	1.032	0.849	1.254	0.755	41.9%
Myocardial infarction	3	1,626/3,225	1.117	0.935	1.334	0.548	60.7%	1.034	0.885	1.207	0.678	12.8%	0.858	0.677	1.087	0.205	59.6%
Based on genotyping method																	
NON-RFLP	6	2,085/3,620	1.171	0.988	1.388	0.069	72.2%	1.271	1.009	1.599	** 0**.041*	64.3%	1.085	0.903	1.303	0.384	29.7%
PCR-RFLP	5	2,258/2,912	0.935	0.804	1.088	0.384	65.8%	0.973	0.773	1.226	0.818	65.5%	0.824	0.696	0.975	** 0**.024*	25.0%
Based on source of control																	
HB	6	1,842/1,643	1.054	0.890	1.247	0.545	61.0%	1.129	0.929	1.373	0.222	36.5%	1.000	0.759	1.317	1.000	53.2%
PB	5	2,501/4,889	1.065	0.884	1.283	0.509	83.8%	1.140	0.863	1.505	0.357	82.2%	0.930	0.779	1.110	0.420	47.1%
Based on sample size of case																	
>=500	5	2,789/4,475	0.991	0.873	1.124	0.885	67.8%	1.059	0.874	1.283	0.560	65.1%	0.897	0.771	1.043	0.158	35.7%
<500	6	1,554/2,057	1.146	0.893	1.472	0.284	79.5%	1.199	0.869	1.655	0.269	73.5%	1.111	0.809	1.525	0.514	55.4%
Based on sample size of control																	
>=500	5	2,783/5,224	0.929	0.853	1.013	0.094	37.9%	0.962	0.854	1.083	0.520	14.7%	0.850	0.740	0.977	** 0**.022*	33.5%
<500	6	1,560/1,288	1.247	1.022	1.521	** 0**.030*	62.9%	1.350	1.025	1.777	** 0**.032*	61.1%	1.212	0.989	1.485	0.064	0.0%
Based on gender ratio of case																	
M/F >=1	8	2,768/4,092	1.108	0.935	1.313	0.236	76.4%	1.212	0.973	1.509	0.087	68.4%	0.974	0.801	1.184	0.791	43.7%
M/F < 1	1	418/421	1.159	0.957	1.404	0.131	-	1.178	0.865	1.605	0.298	-	1.281	0.922	1.779	0.140	-
NA	2	1,157/2,019	0.890	0.803	0.987	** 0**.027*	0.0%	0.872	0.738	1.031	0.110	0.0%	0.838	0.705	0.995	** 0**.044*	0.0%
Based on gender ratio of control																	
M/F >=1	6	2,092/3,344	1.115	0.882	1.410	0.363	82.9%	1.226	0.909	1.654	0.182	77.4%	0.949	0.732	1.231	0.694	52.9%
M/F < 1	3	1,094/1,169	1.115	0.992	1.254	0.067	0.0%	1.180	0.979	1.423	0.083	0.0%	1.141	0.933	1.395	0.200	0.0%
NA	2	1,157/2,019	0.890	0.803	0.987	**0**.027*	0.0%	0.872	0.738	1.031	0.110	0.0%	0.838	0.705	0.995	** 0**.044*	0.0%
Based on mean age of case																	
>=55	5	2,189/4,334	0.958	0.853	1.076	0.469	54.3%	0.996	0.882	1.126	0.953	0.0%	0.880	0.710	1.089	0.239	60.8%
<55	4	939/658	1.328	0.944	1.869	0.103	71.9%	1.431	0.925	2.213	0.107	70.4%	1.260	0.928	1.710	0.139	0.0%
NA	2	1,215/1,540	0.985	0.830	1.169	0.862	60.7%	1.001	0.714	1.404	0.995	73.0%	0.951	0.797	1.136	0.582	0.0%
Based on mean age of control																	
>=55	4	1,986/4,125	0.944	0.828	1.077	0.393	62.6%	0.981	0.863	1.115	0.768	0.0%	0.868	0.678	1.111	0.260	69.9%
<55	5	1,142/867	1.267	0.970	1.657	0.083	68.9%	1.386	0.982	1.956	0.064	65.0%	1.171	0.904	1.516	0.231	0.0%
NA	2	1,215/1,540	0.985	0.830	1.169	0.862	60.7%	1.001	0.714	1.404	0.995	73.0%	0.951	0.797	1.136	0.582	0.0%

PCR-RELP, polymerase chain reaction-restriction fragment length polymorphisms; HB, hospital-based; PB, population-based; W, wild allele; M, mutant allele; WW, wild homozygote; WM, heterozygote; MM, mutant homozygote; HWE, Hardy-Weinberg Equilibrium; CI, confidence interval; LL, lower limit; UL, upper limit; NA, not available.

Bolded values indicate *P* < 0.05.

* *P *< 0.05.

**Figure 3:**
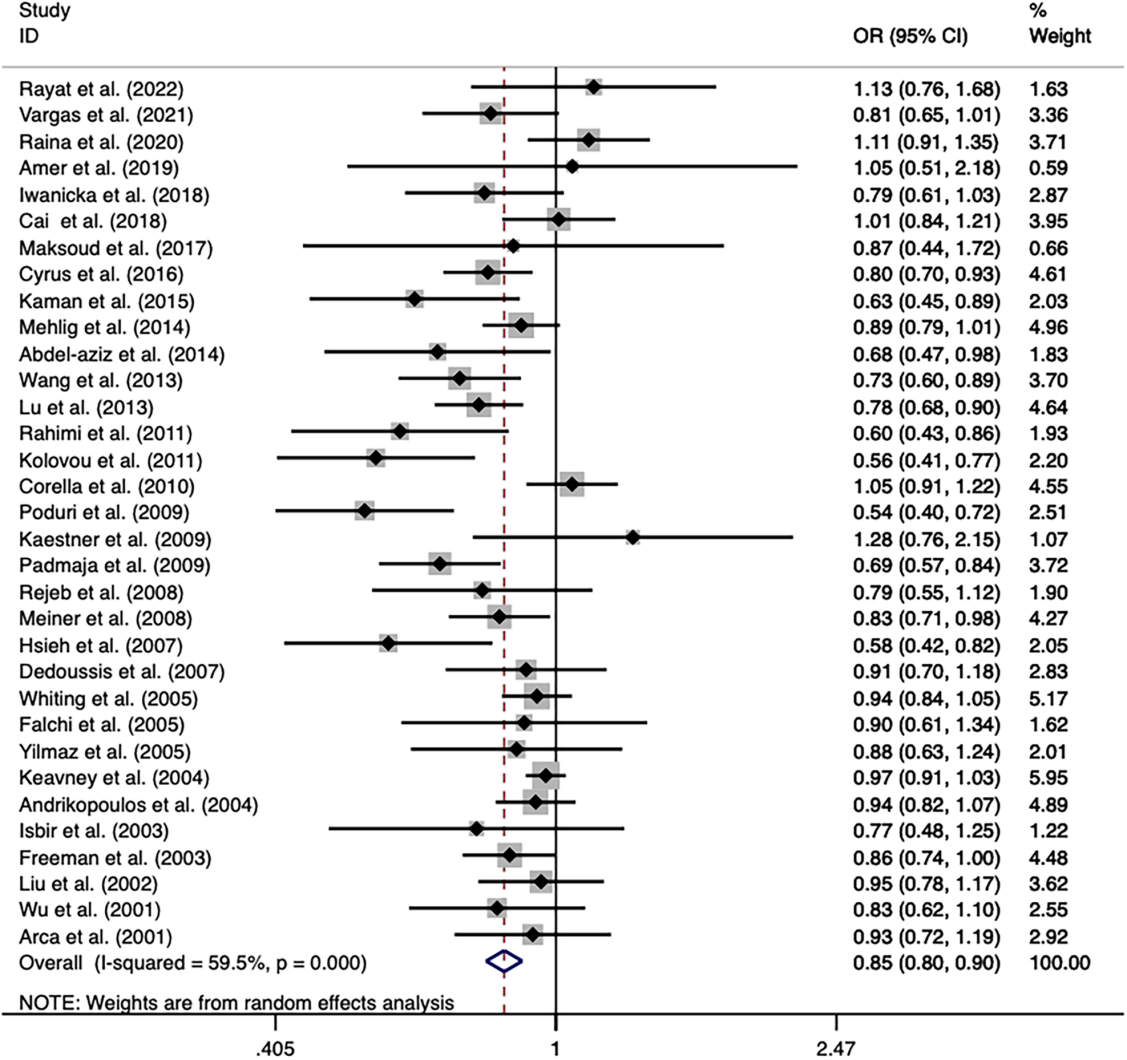
Forest plot of the meta-analysis between the *CETP* rs708272 polymorphism and CAD under the allele model (Avs.G).

The overall analyses of all the 33 studies on rs708272 indicated that the carriers of the allele A were significantly associated with a reduced risk of CAD than the non-carriers under the allele model (OR = 0.846, 95% CI = 0.798–0.897, *P* <* *0.001) (Figure 3), the dominant model (OR=0.838, 95% CI = 0.769–0.913, *P* <* *0.001) and the recessive model (OR=0.758, 95% CI = 0.687–0.836, *P < *0.001). Since a moderate heterogeneity was observed under the allele model (*I^2^*^ ^=* *59.5%, *P < *0.001), the dominant model (*I^2 ^*=* *57.5%, *P < *0.001), and the recessive model (*I^2^*^ ^=* *51.4%, *P* <* *0.001), the random effects model was used.

After the exclusion of the studies deviating from HWE in the control, the statistical significance of the analysis did not substantially change. Subgroup analysis based on ethnicity identified a significance of association of the *CETP* rs708272 polymorphism with CAD both in Caucasians and Asians. The allele rs708272-A based on CAD subtypes significantly reduced the risk of myocardial infarction under the allele model (OR=0.849, *P *=* *0.032) and recessive model (OR = 0.874, *P = *0.005), but not under the dominant model (OR = 0.965, *P *=* *0.361). Substantial heterogeneity in the coronary stenosis subgroup under the three genetic models (*I^2 ^*= 62.0%, *I^2 ^= *60.2%, *I^2 ^*=* *56.5%) was not observed in the myocardial infarction subgroup (*I^2 ^*= 0.0% for all). Table 3 also shows significant associations in NON-RFLP, RFLP, HB, and PB subgroups (all *P < *0.05). Based on the patient sample size, the risk estimation in studies with large samples (size ≥ 500) appeared relatively conserved under the allele model (OR = 0.907, *P = *0.006), dominant model (OR = 0.883, *P = *0.02) and recessive model (OR = 0.831, *P = *0.001), while the risk estimation in studies with small samples (size < 500) was slightly overestimated. Based on the size of the control, the heterogeneity of both studies with large samples (size ≥ 500) and small samples (size < 500) decreased under the allele model (*I^2 ^*= 49.7%, *I^2 ^*= 57.9%) and dominant model (*I^2 ^*= 20.5%, *I^2 ^*= 51.4%), suggesting that the heterogeneity might originate from this factor. Based on the gender ratio, the *CETP* rs708272 polymorphism was found to have a significant association with CAD under the three genetic models (all *P* < 0.05) in the cases and controls where males constituted the majority. However, in the cases where females predominated, this SNP was not significantly associated with CAD under the allele model (OR = 0.786, *P* = 0.091), dominant model (OR = 0.784, *P* = 0.360), or recessive model (OR = 0.668, *P* = 0.006). This non-association was also found in the female-dominated controls under the dominant model (OR = 0.818 *P* = 0.075). Based on the mean age of cases/controls, the significant association of this SNP with CAD was observed in all age groups under the three genetic model (all *P *< 0.05), and this finding was consistent with the overall analysis.

**Figure 4:**
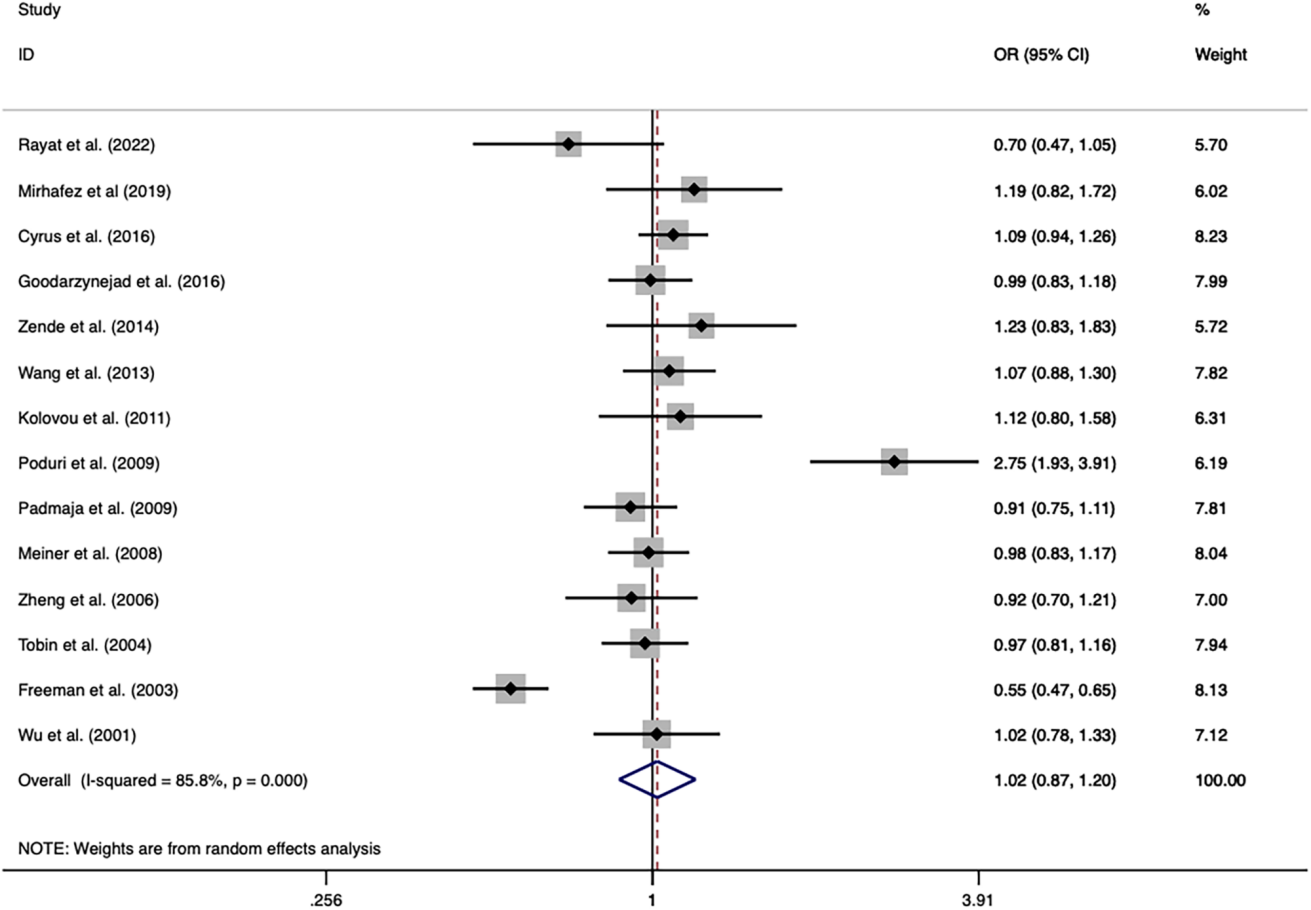
Forest plot of the meta-analysis between the *CETP* rs5882 polymorphism and CAD under the allele model (Avs.G).

**Figure 5:**
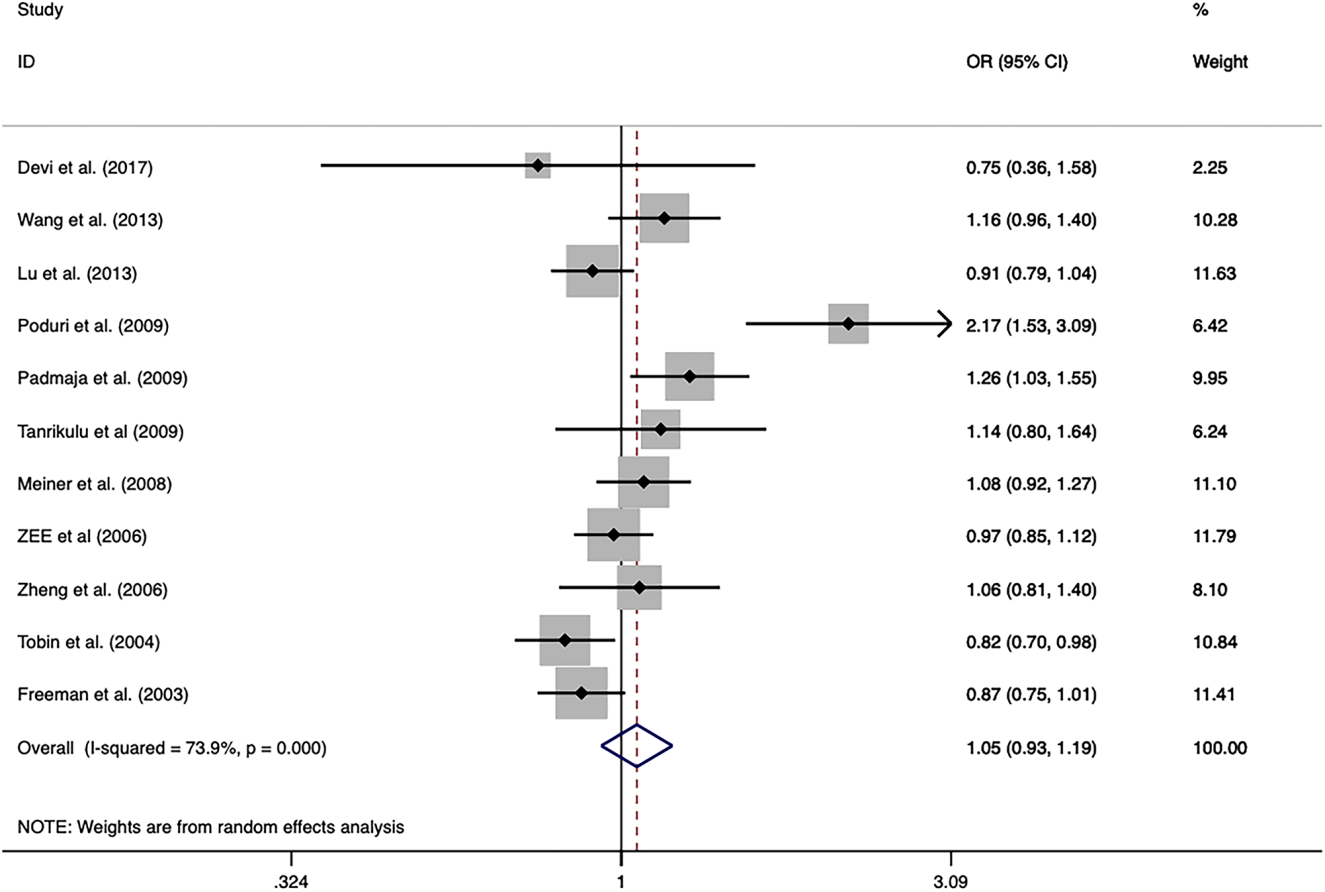
Forest plot of the meta-analysis between the *CETP* rs1800775 polymorphism and CAD under the allele model (Avs.C).

The overall analysis indicated that the *CETP* rs5882 and rs180075 polymorphisms were not significantly associated with CAD under the allele model (OR = 0.846, *P* < 0.001), dominant model (OR = 0.838, *P* < 0.001) and recessive model (OR = 0.758, *P* < 0.001) (Figures 4, 5), accompanied by a significant heterogeneity. This result contradicted the 2008 review mentioned earlier, which suggested that these two SNPs were associated with CAD.

The exclusion of the studies with deviations from HWE in the control group did not substantially alter the statistical significance of the analysis. The subgroup analysis on the rs5882 polymorphism suggested that it was associated with an increased risk of CAD under the dominant model (OR = 1.288, 95% CI = 1.037–1.599, *P *= 0.022) in the NON-RFLP subgroup. However, all the other subgroups except this one showed that the *CETP* rs5882 polymorphism was not significantly associated with CAD under the three genetic models. According to genotyping methods, the *CETP* rs180075 polymorphism was associated with an increased risk of CAD under the dominant model (OR = 1.271, 95% CI = 1.009–1.599, *P *= 0.041) in the NON-RFLP subgroup, with reduced risk of CAD under the recessive model (OR = 0.824, 95% CI = 0.696–0.975, *P* = 0.024) in the PCR-RFLP subgroup. According to the sample size of the control, the *CETP* rs180075 polymorphism was associated with an increased risk of CAD under the allele model (OR = 1.247, 95% CI = 1.022–1.521, *P *= 0.03) and the dominant model (OR=1.35, 95% CI = 1.025–1.777, *P* = 0.032) in studies using small samples (size < 500). On the contrary, the *CETP* rs180075 polymorphism was associated with a reduced risk of CAD under the recessive model (OR = 0.85, 95% CI = 0.74–0.977, *P* = 0.022) in studies using large samples (size ≥ 500). The heterogeneity decreased in studies with both large samples (size ≥ 500) and small samples (size < 500) under the three genetic models, suggesting that the heterogeneity might be attributed to variations in sample size.

### Association of the five uncommon *CETP gene* polymorphisms with CAD

 Table 4 shows no significant associations of *CETP* rs4783961, rs247616, rs5883, rs1800776, and rs1532624 polymorphisms with CAD under the three genetic models. The results might be more susceptible to random factors due to the limited sample size, which might contribute to an increased heterogeneity. Subgroup analysis was not performed due to the lack of a sufficient number of studies.

**Table 4:** Overall analyses of the association of the three uncommon *CETP* gene polymorphisms with CAD.

Subgroups	Studies (*n*)	Case/Control (*n*/*n*)	Allele Model (M vs. W)						Dominant Model (MM + WM vs. WW)						Recessive Model (MM vs. WW + WM)					
			OR	95% CI		*P* value	*I* ^2^	Heterogeneity	OR	95% CI		*P* value	*I* ^2^	Heterogeneity	OR	95% CI		*P* value	*I* ^2^	Heterogeneity
				LL	UL			*P* value		LL	UL			*P* value		LL	UL			*P* value
rs4783961 (G>A)	3	1,552/1,964	1.066	0.833	1.363	0.614	81.5%	0.004	1.137	0.787	1.643	0.494	80.7%	0.006	0.977	0.663	1.440	0.908	72.2%	0.027
rs247616 (C>T)	3	918/696	0.892	0.630	1.262	0.518	77.0%	0.013	0.813	0.489	1.350	0.423	82.1%	0.004	0.921	0.629	1.347	0.671	0.0%	0.593
rs5883 (C>T)	2	368/345	1.288	0.681	2.434	0.436	0.0%	0.976	1.298	0.680	2.479	0.429	0.0%	0.983	–	–	–	–	–	–
rs1800776 (C>A)	2	658/648	1.162	0.945	1.429	0.634	0.0%	<0.001	1.134	0.909	1.415	0.778	0.0%	<0.001	2.465	0.909	6.679	0.797	0.0%	<0.001
rs1532624 (C>A)	2	1,913/1,612	0.862	0.733	1.015	0.402	0.0%	<0.001	0.778	0.552	1.095	0.157	50.2%	0.0319	0.852	0.602	1.207	0.981	0.0%	<0.001

PCR-RELP, polymerase chain reaction-restriction fragment length polymorphisms; HB, hospital-based; PB, population-based; W, wild allele; M, mutant allele; WW, wild homozygote; WM, heterozygote; MM, mutant homozygote; HWE, Hardy-Weinberg Equilibrium; CI, confidence interval; LL, lower limit; UL, upper limit.

* *P* < 0.05.

### Association of the *CETP rs708272* polymorphism with CETP level and lipid serum concentrations

**Figure 6:**
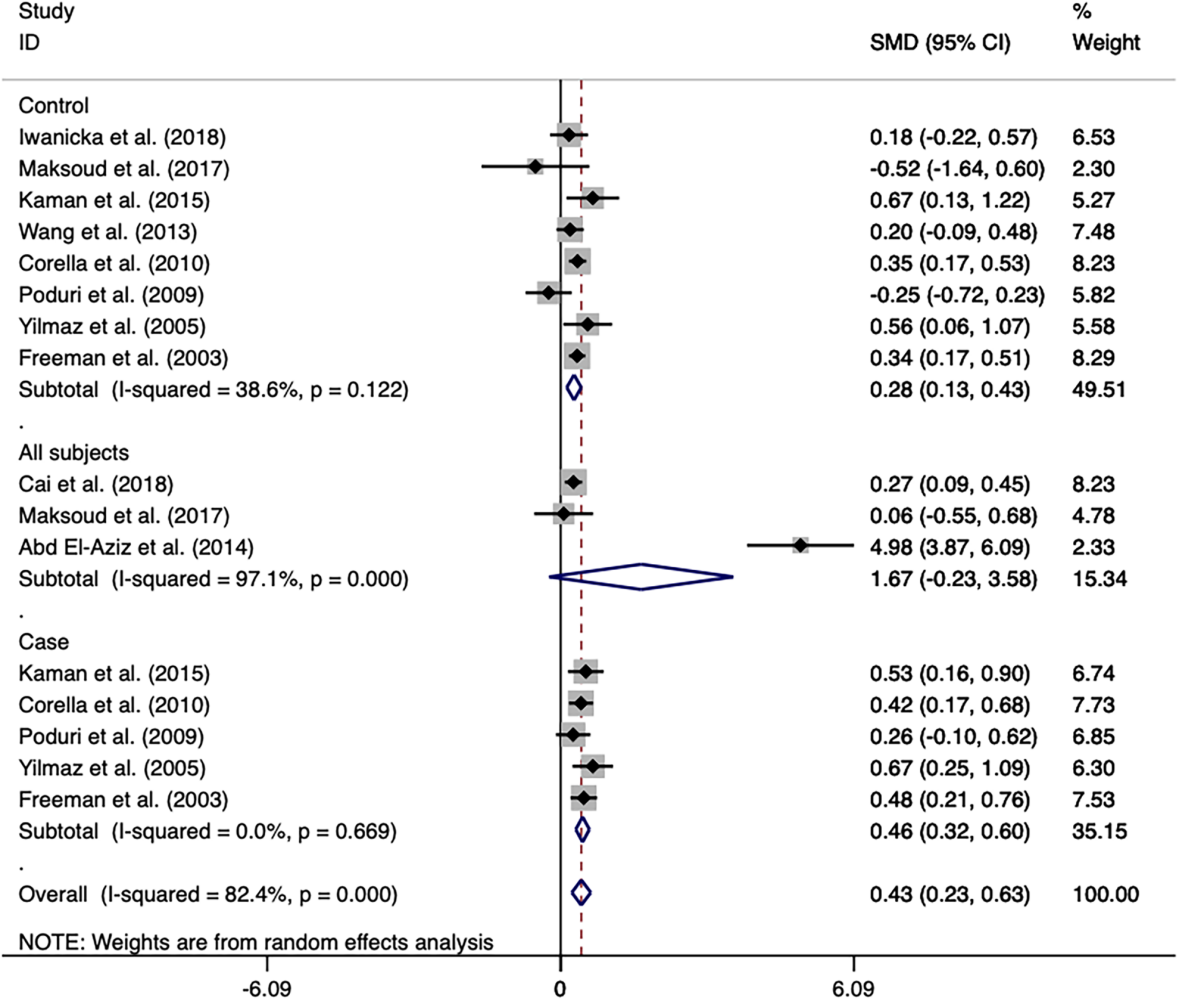
Forest plot of the meta-analysis between the *CETP* rs708272 polymorphism and HDL-C concentrations under the homozygote model (AAvs.GG).

A meta-analysis was performed after the extraction of the data of CETP and lipid levels to find the association. Table 5 shows which information should be extracted using HDL-C as an example. Table 6 shows the association of the *CETP* rs708272 polymorphism with CETP level and HDL-C, LDL-C, TG, and TC concentrations in the patients, control, and all subjects under the homozygote model and the heterozygote model. AA genotype had higher HDL-C concentrations compared with the GG genotype in the case group (SMD = 0.459, *P *<* *0.001) and control group (SMD = 0.279, *P *<* *0.001) without significant heterogeneity (*I^2 ^*= 0.0%, *I^2 ^*= 40.6%) (Figure 6). Similarly, the GA genotype had higher HDL-C concentrations than the GG genotype in the patient group (SMD = 0.216, *P < *0.001) and control group (SMD = 0.197, *P *<* *0.001) without significant heterogeneity (*I^2 ^*= 20.6%, *I^2^*^ ^= 0.0%). The overall analysis indicated that the AA genotype and GA genotype were statistically significant compared with the GG genotype, with considerable heterogeneity (*I^2^* = 83.0%, *I*^2^ = 74.0%) which might result from the types of groups. In conclusion, the carriers of allele A had higher HDL-C concentrations than the non-carriers. HDL particles play a pivotal role in removing excess cholesterol from peripheral tissues, reducing inflammation, protecting the endothelium, and preventing oxidative damage—all of which contribute to their anti-atherogenic properties. Higher levels of HDL in the blood are generally associated with a lower risk of atherosclerosis and cardiovascular disease. No significant correlation was found between the *CETP* rs708272 polymorphism and TC, TG, and LDL-C across the studied groups under two genetic models.

**Table 5:** Selected studies including the data of the HDL-C concentrations across the *CETP* rs708272 polymorphism genotypes.

First author	Year	Group	AA			GA			GG		
			Mean(mg/dl)	SD	*n*	Mean(mg/dl)	SD	*n*	Mean(mg/dl)	SD	*n*
Iwanicka et al. (19)	2,018	Control	56.46	22.82	36	58.01	22.82	123	52.98	18.17	80
Cai et al. (20)	2018	All subjects	45.24	11.99	185	42.92	10.83	442	42.15	11.21	343
Maksoud et al. (21)	2017	Control	30.47	11.06	5	47.87	17.34	16	37.86	14.25	9
		All subjects	42.43	16.28	14	43.84	13.77	47	41.63	10.49	39
Kaman et al. (23)	2015	Case	45.52	10.81	44	40.99	10.89	81	40.38	9.12	85
		Control	51.93	9.47	29	44.19	8.85	45	45.34	9.93	26
Abd El-Aziz et al. (25)	2014	All subjects	58.90	7.52	18	44.20	8.18	60	31.32	4.19	38
Wang et al. (26)	2013	Control	56.46	27.07	74	56.46	23.98	207	51.82	21.27	139
Corella et al. (30)	2010	Case	54.00	17.60	86	49.30	13.30	247	47.70	13.60	224
		Control	56.60	14.40	161	54.90	15.50	537	51.70	13.70	482
Poduri et al. (31)	2009	Case	36.63	10.12	41	35.86	7.66	107	34.00	9.96	117
		Control	38.41	7.55	35	42.68	10.08	82	40.24	7.07	33
Yilmaz et al. (38)	2005	Case	44.50	13.50	35	41.40	8.80	72	37.60	8.00	66
		Control	45.00	14.20	26	35.80	10.40	46	37.10	13.60	39
Freeman et al. (44)	2003	Case	43.31	8.51	76	42.15	8.89	259	39.44	7.73	164
		Control	46.02	9.67	225	44.47	9.28	541	42.54	10.44	339

SD, standard deviation.

**Table 6:** Overall and subgroup analyses of the association of the *CETP* rs708272 polymorphisms with CETP and lipids levels.

Lipids	Group	Studies (*n*)	Sizes (*n*)	Homozygote Model					Heterozygote Model				
				SMD	95% CI		*P* value	*I* ^2^	SMD	95% CI		*P* value	*I* ^2^
				* *	LL	UL				LL	UL		
HDL-C	Overall	16	6,225	0.435	0.235	0.635	**<0**.001*	83.00%	0.204	0.149	0.26	**<0**.001*	74.40%
	All subjects	3	1,118	1.701	−0.239	3.640	0.086	97.20%	0.207	0.078	0.336	**0**.002*	95.90%
	Case	5	1,704	0.459	0.317	0.600	**<0**.001*	0%	0.216	0.111	0.321	**<0**.001*	20.60%
	Control	8	3,335	0.279	0.124	0.433	**<0**.001*	40.60%	0.197	0.197	0.274	**<0**.001*	0.00%
LDL-C	Overall	13	4,201	0.026	−0.064	0.115	0.576	5.60%	0.046	−0.021	0.113	0.182	25.10%
	All subjects	3	1,186	0.110	−0.055	0.274	0.191	0.00%	0.116	−0.011	0.243	0.074	0.00%
	Case	4	1,205	−0.112	−0.275	0.274	0.178	0.00%	−0.040	−0.164	0.084	0.525	0.00%
	Control	6	1,801	0.066	−0.075	0.207	0.358	37.40%	0.058	−0.044	0.161	0.262	47.60%
TG	Overall	11	3,786	0.026	−0.226	0.277	0.842	81.70%	0.106	−0.214	0.246	0.893	87.80%
	All subjects	5	1,186	0.224	−0.47	0.918	0.527	85.90%	0.242	−0.27	0.754	0.354	86.90%
	Case	3	940	−0.031	−0.215	0.152	0.738	0.00%	−0.122	−0.327	0.083	0.244	44.10%
	Control	3	1,421	−0.106	−0.348	0.135	0.387	38.00%	−0.053	−0.215	0.109	0.522	25.80%
TC	Overall	13	4,201	−0.07	−0.159	0.020	0.126	30.10%	−0.027	−0.094	0.040	0.424	30.60%
	All subjects	3	1,186	−0.088	−0.252	0.076	0.293	29.60%	−0.076	−0.203	0.051	0.240	7.00%
	Case	4	1,205	−0.100	−0.264	0.063	0.228	0.00%	−0.029	−0.153	0.095	0.649	19.40%
	Control	6	1,541	−0.034	−0.175	0.107	0.639	60.40%	0.005	−0.097	0.107	0.921	52.20%
CETP	Case	1	210	−0.485	−0.854	−0.116	** 0**.010*	0.00%	0.012	−0.292	0.316	0.939	0.00%

LDL-C, low-density lipoprotein cholesterol; HDL-C; high-density lipoprotein cholesterol; TG, triglycerides; TC, total cholesterol.

Bolded values indicate *P* < 0.05.

* *P* < 0.05.

In the patient group, the AA genotype had a lower CETP level (SMD = −0.485, *P* = 0.010) compared with the GG genotype. However, this conclusion is not reliable due to the limited sample size and the presence of only one study.

### Meta-regression analysis

The subgroup analysis of rs708272 and rs1800775 indicated that the heterogeneity might be due to the sample size which was equivalent to the sum of each genotype count. Univariate and multivariate meta-regression analyses, including three types of genotype counts of case/control, were conducted to find potential sources of heterogeneity in the studies on the association of *CETP* gene polymorphism with CAD. As regards rs708272, the heterogeneity could be explained by GG genotype counts of the patients and AA and GG genotype counts of the control under the allele model (*P *=* *0.027, *P* =* *0.004, *P *=* *0.041). AA and GA genotype counts of the patients and AG genotype counts of the control could explain the source of heterogeneity under the recessive model (*P* =* *0.002, *P *=* *0.001, *P *=* *0.002). AA and GA genotype counts of the case/control could explain the source of heterogeneity under the dominant model (*P *=* *0.003, *P *=* *0.025, *P* =* *0.001, *P *=* *0.049). As regards rs5882, the heterogeneity could be explained by the GG genotype counts of the control under the allele model (*P* = 0.012). The meta-regression analysis failed to find the source of heterogeneity under the dominant model and the recessive model. As regards rs1800775, the source of heterogeneity was explained by the AA and CA genotype counts of the patients under the recessive model (*P* = 0.007, *P* = 0.019). The source of heterogeneity was explained by the AA and CA genotype counts of the patients and CC genotype counts of the control under the allele model (*P* = 0.008, *P* = 0.032, *P *= 0.010). The source of heterogeneity was explained by the CC genotype counts of the control under the allele model (*P* = 0.008).

### Sensitivity analysis and publication bias

**Figure 7:**
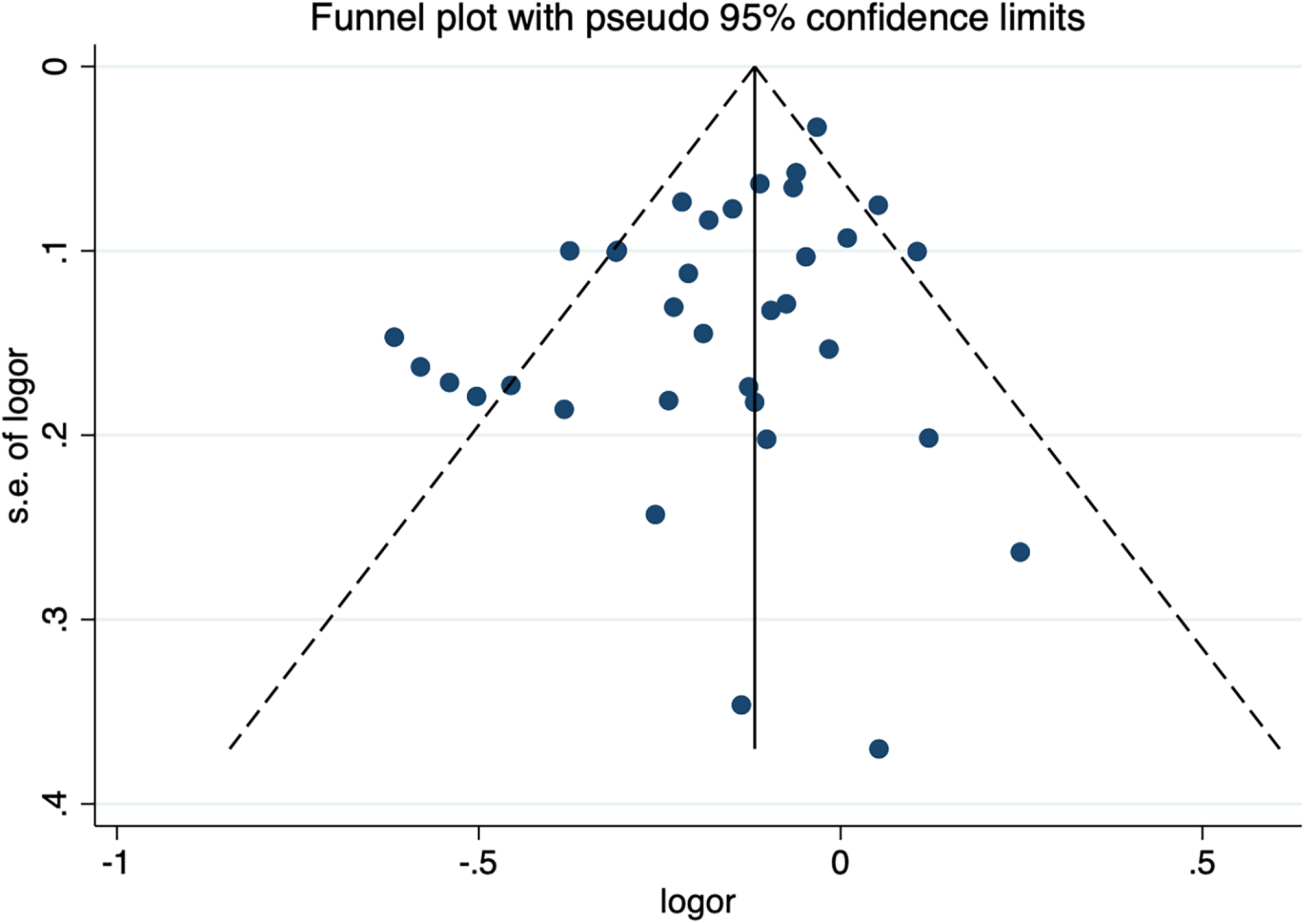
Funnel plot of the meta-analysis between the *CETP* rs708272 polymorphism and CAD under the allele model (Avs.G).

**Figure 8:**
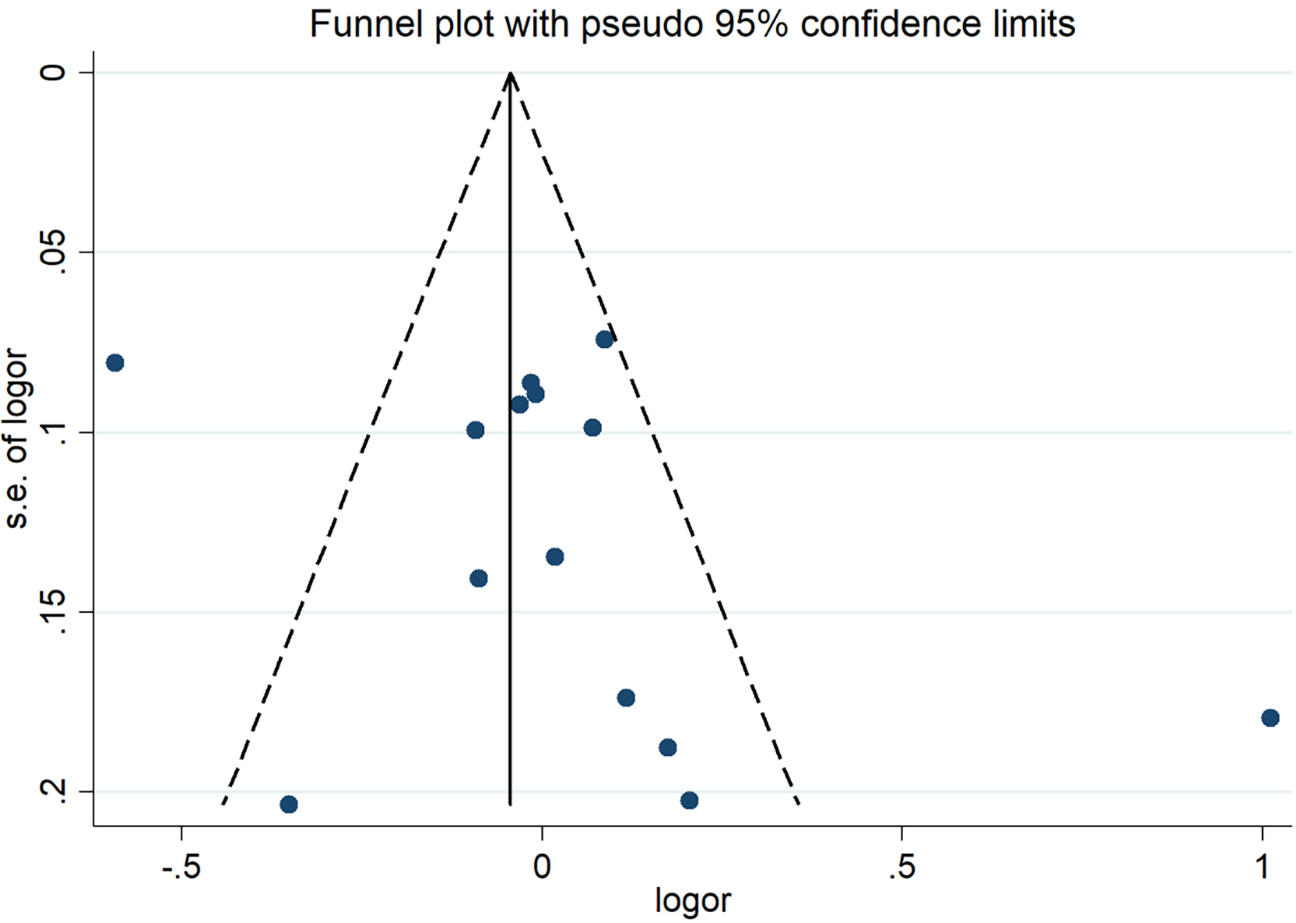
Funnel plot of the meta-analysis between the *CETP* rs5882 polymorphisms and CAD under the allele model (Avs.G).

**Figure 9:**
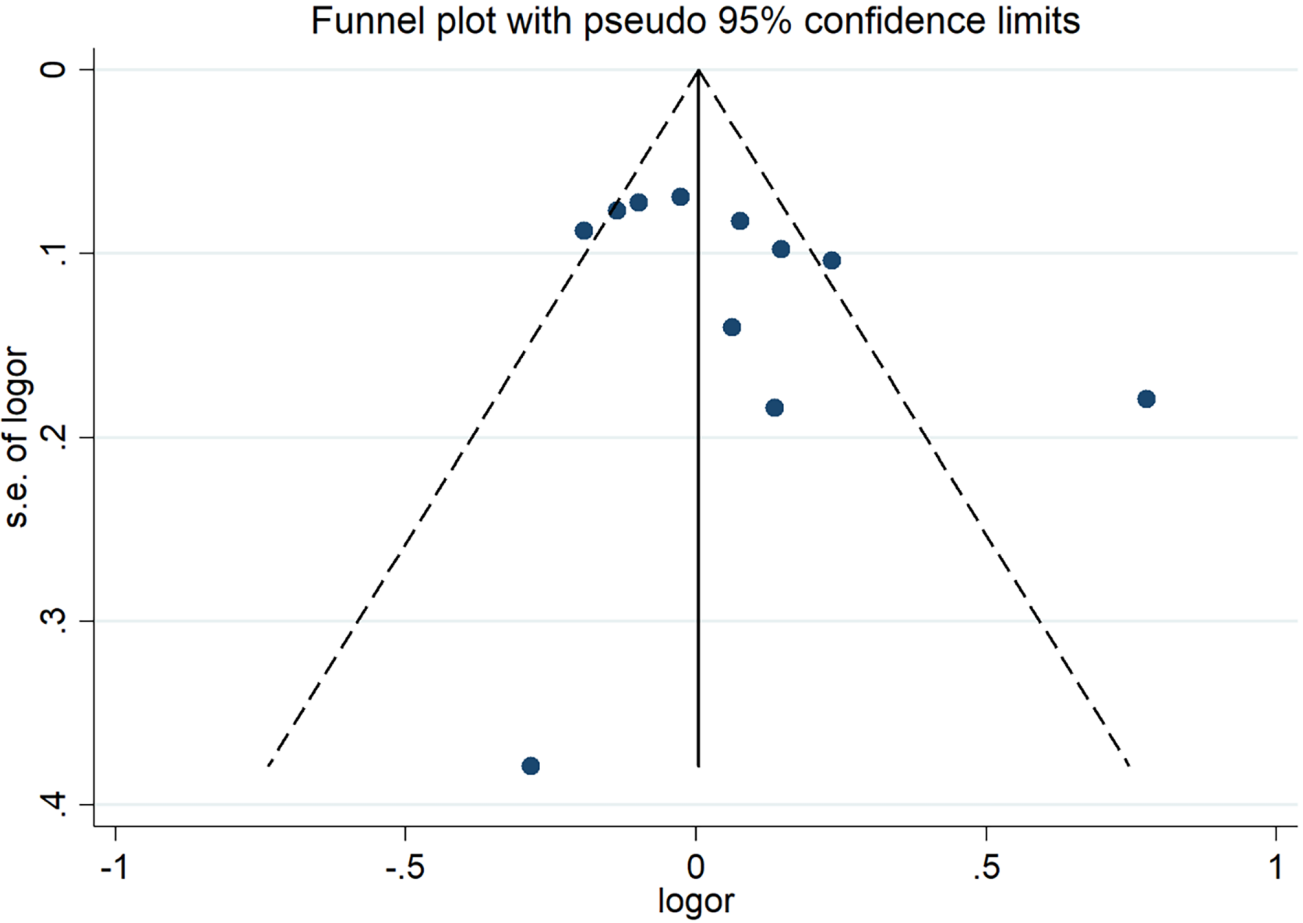
Funnel plot of the meta-analysis between the *CETP* rs1800775 polymorphism and CAD under the allele model (Avs.C).

The sensitivity analysis of the association between the *CETP* gene polymorphism and CAD indicated that no single study affected the overall OR and the statistical significance under the three genetic models. As regards the rs708272 polymorphism, the funnel plot showed an imbalance between the number of data points on the left and right sides, resulting in an asymmetrical appearance (Figure 7). The Egger's test confirmed a remarkable publication bias under the allele model (*P *=* *0.014), dominant model (*P *=* *0.035), and recessive model (*P* =* *0.02). The Begg's test did not detect any publication bias. The trim-and-fill method did not trim any studies for imputation. The Egger's test revealed the disappearance of publication bias under the allele model (*P =* 0.190) and the dominant model (*P =* 0.504), but not under the recessive model (*P *= 0.045) after excluding studies with significant departure from HWE. As regards the rs5882 polymorphism and rs1800775 polymorphism, the shapes of the funnel plots did not show any evident asymmetry (Figures 8, 9). Egger's test confirmed no publication bias under the allele model (*P* = 0.191, *P* = 0.173), the dominant model (*P* = 0.298, *P* = 0.400), and the recessive model (*P *= 0.097, *P *= 0.138).

**Figure 10:**
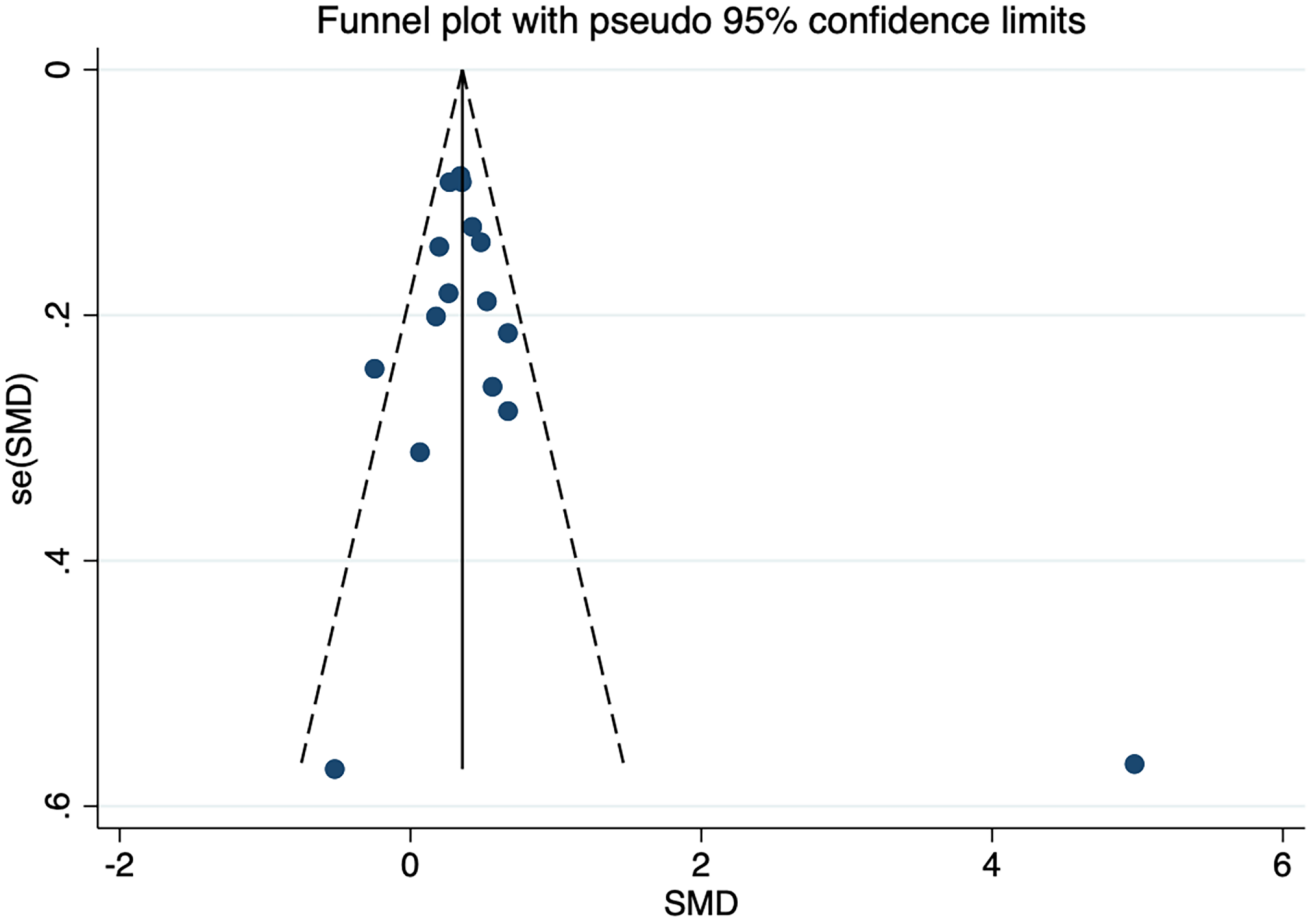
Funnel plot of the meta-analysis between the *CETP* rs708272 polymorphism and HDL-C concentrations under the homozygote model (AAvs.GG).

No individual studies substantially influenced the overall SMD when associated with HDL-C. The funnel plots were almost symmetrical (Figure 10). Egger's test and Begg's test did not find any publication bias. The inclusion of studies investigating the association of the *CETP* rs708272 polymorphism with other lipid serum concentrations did not have any significant impact on the overall SMD. The funnel plots showed nearly symmetrical distribution of the studies. Egger's test and Begg's test did not detect any evidence of publication bias.

## Discussion

In the present meta-analysis, our findings suggested that carriers of allele A of the *CETP* rs708272 polymorphism had higher HDL-C concentrations, lower CETP levels, and lower risk of CAD than no-carries. However, the reliability of the association between the *CETP* rs708272 polymorphism and CETP level was questionable considering the restricted sample size and the absence of additional studies. HDL particles exhibit a diverse range of atheroprotective activities including the efflux of cellular cholesterol, reduction of the inflammatory responses, and protection against pathological oxidation (61). Therefore, the potential mechanism underlying the observed association between the *CETP* rs708272 polymorphism and a reduced risk of CAD might be due to the increase of HDL-C concentrations attributed to the rs708272 G-to-A mutation. Based on the findings mentioned above, our speculation was that CETP inhibitors might prevent the development of CAD. Although some studies showed promising effects, including a modest increase in HDL cholesterol levels and a potential reduction in LDL cholesterol and triglyceride levels, the translation of these lipid-modifying effects into significant improvements in cardiovascular outcomes such as the decrease in the incidence of CAD has been inconsistent (62–65). It is possible that HDL-C concentrations may not fully reflect the functionality and diversity of HDL particles, since they primarily served as a general measure of cholesterol carried by HDL. Subgroup analyses was evaluated to examine the potential effects of CAD sub-type, genotyping techniques, source of control, sample size, gender, and age on the connection between genetic polymorphisms and CAD. The findings of the subgroup analysis revealed a significant association between the *CETP* rs708272 polymorphism and a decreased risk of CAD in almost all subgroups. It's worth noting that in case and control groups predominantly composed of females, no significant association was found. However, due to the limited number of studies and small sample sizes, these conclusions are not reliable. During the analysis of articles, we observed that the majority of studies include case and control groups with a significantly higher number of males than females, and gender differences were not further analyzed. This suggests that future researchers should pay more attention to the impact of gender.

However, no significant association of the *CETP* rs5882 and rs1800775 polymorphisms with the risk of CAD was found, which was in contradiction with the finding reported by the previous review. Subgroup analysis revealed no significant association between these two SNPs and CAD in most subgroups. It was worth noting that rs1800775 polymorphism was associated with a reduced risk of CAD in studies using large samples, while it was associated with an increased risk of CAD in studies using small samples. More accurate results might be obtained including more studies with larger sample sizes.

No significant correlation was identified between the five uncommon *CETP* gene polymorphisms and CAD. However, the reliability of these results was limited due to the inclusion of only 2–3 studies for each SNP.

SNPs are closely associated with the occurrence and progression of many diseases (66). The association between SNPs and specific diseases might help identifying the role of genetic factors in disease development, providing a basis for an early diagnosis, prevention, and personalized treatment (67). This meta-analysis revealed a significant association between the *CETP* rs708272 polymorphism and CAD. Furthermore, it is worth considering the potential for early intervention strategies aimed at individuals carrying the *CETP* rs708272 risk allele, with the goal of preventing CAD. Our results might help in the determination of disease etiology, as well as improve early intervention to delay or prevent the onset of CAD.

Our current meta-analysis has certain potential limitations. Firstly, studies published in English were the only included, resulting in an insufficient sample size and a limited number of studies for some SNPs. Secondly, a significant heterogeneity was found in our study, potentially compromising the robustness of the findings. Thirdly, only the relationship between each SNP and CAD was investigated due to the lack of sufficient raw data, without examining the interactions between SNPs. While these limitations are acknowledged, it is also prudent to consider the potential impact of publication bias. Publication bias, whereby studies with significant or positive findings are more likely to be published, could introduce a bias in our meta-analysis results. Although we made efforts to minimize this bias through a comprehensive literature search, it remains a potential limitation that should be recognized.

## Conclusion

The *CETP* rs708272 polymorphism is significantly associated with a lower risk of CAD and higher HDL-C concentration. However, the *CETP* rs5882 and rs1800775, rs4783961, rs247616, rs5883, rs1800776, and rs1532624 do not show any significant association with CAD.

## Data Availability

The original contributions presented in the study are included in the article/Supplementary Material, further inquiries can be directed to the corresponding author.

## References

[1] Tsao CW Aday AW Almarzooq ZI Anderson CAM Arora P Avery CL Heart disease and stroke statistics-2023 update: a report from the American heart association. Circulation. (2023) 147 (8):e93–e621. 10.1161/CIR.0000000000001123 36695182 PMC12135016

[2] Lanktree MB Hegele RA. Gene-gene and gene-environment interactions: new insights into the prevention, detection and management of coronary artery disease. Genome Med. (2009) 1(2):28. 10.1186/gm28 19341499 PMC2664961

[3] Yusuf S Hawken S Ounpuu S Dans T Avezum A Lanas F Effect of potentially modifiable risk factors associated with myocardial infarction in 52 countries (the INTERHEART study): case-control study. Lancet. (2004) 364(9438):937–52. 10.1016/S0140-6736(04)17018-9 15364185

[4] de Grooth GJ Klerkx AH Stroes ES Stalenhoef AF Kastelein JJ Kuivenhoven JA. A review of CETP and its relation to atherosclerosis. J Lipid Res. (2004) 45(11):1967–74. 10.1194/jlr.R400007-JLR200 15342674

[5] Sirtori CR Corsini A Ruscica M. The role of high-density lipoprotein cholesterol in 2022. Curr Atheroscler Rep. (2022) 24(5):365–77. 10.1007/s11883-022-01012-y 35274229 PMC8913032

[6] Papp AC Pinsonneault JK Wang D Newman LC Gong Y Johnson JA Cholesteryl ester transfer protein (CETP) polymorphisms affect mRNA splicing, HDL levels, and sex-dependent cardiovascular risk. PLoS One. (2012) 7(3):e31930. 10.1371/journal.pone.0031930 22403620 PMC3293889

[7] Drayna D Lawn R. Multiple RFLPs at the human cholesteryl ester transfer protein (CETP) locus. Nucleic Acids Res. (1987) 15(11):4698. 10.1093/nar/15.11.4698 2884631 PMC340900

[8] Boekholdt SM Thompson JF. Natural genetic variation as a tool in understanding the role of CETP in lipid levels and disease. J Lipid Res. (2003) 44(6):1080–93. 10.1194/jlr.R200018-JLR200 12639975

[9] Dachet C Poirier O Cambien F Chapman J Rouis M. New functional promoter polymorphism, CETP/-629, in cholesteryl ester transfer protein (CETP) gene related to CETP mass and high density lipoprotein cholesterol levels: role of Sp1/Sp3 in transcriptional regulation. Arterioscler Thromb Vasc Biol. (2000) 20(2):507–15. 10.1161/01.ATV.20.2.507 10669650

[10] Thompson A Di Angelantonio E Sarwar N Erqou S Saleheen D Dullaart RP Association of cholesteryl ester transfer protein genotypes with CETP mass and activity, lipid levels, and coronary risk. JAMA. (2008) 299(23):2777–88. 10.1001/jama.299.23.2777 18560005

[11] Moher D Liberati A Tetzlaff J Altman DG. Preferred reporting items for systematic reviews and meta-analyses: the PRISMA statement. PLoS Med. (2009) 6(7):e1000097. 10.1371/journal.pmed.1000097 19621072 PMC2707599

[12] Stang A. Critical evaluation of the Newcastle-Ottawa scale for the assessment of the quality of nonrandomized studies in meta-analyses. Eur J Epidemiol. (2010) 25(9):603–5. 10.1007/s10654-010-9491-z 20652370

[13] Higgins JP Thompson SG Deeks JJ Altman DG. Measuring inconsistency in meta-analyses. Br Med J. (2003) 327(7414):557–60. 10.1136/bmj.327.7414.557 12958120 PMC192859

[14] Peters JL Sutton AJ Jones DR Abrams KR Rushton L. Comparison of two methods to detect publication bias in meta-analysis. JAMA. (2006) 295(6):676–80. 10.1001/jama.295.6.676 16467236

[15] Rayat S Ramezanidoraki N Kazemi N Modarressi MH Falah M Zardadi S Association study between polymorphisms in MIA3, SELE, SMAD3 and CETP genes and coronary artery disease in an Iranian population. BMC Cardiovasc Disord. (2022) 22(1):298. 10.1186/s12872-022-02695-6 35768776 PMC9245199

[16] Vargas-Alarcón G Pérez-Méndez O Posadas-Sánchez R Peña-Duque MA Martínez-Ríos MA Delgadillo-Rodriguez H Los polimorfismos rs4783961 y rs708272 del gen CETP son asociados con la enfermedad arterial coronaria y no con la restenosis tras el implante de un stent coronario. Archivos de Cardiología de México. (2021) 92(3):334–41. 10.24875/ACM.21000039 PMC9262298 34594055

[17] Raina JK Sharma M Panjaliya RK Dogra V Bakaya A Kumar P. Association of ESR1 (rs2234693 and rs9340799), CETP (rs708272), MTHFR (rs1801133 and rs2274976) and MS (rs185087) polymorphisms with coronary artery disease (CAD). BMC Cardiovasc Disord. (2020) 20(1):340. 10.1186/s12872-020-01618-7 32682401 PMC7368753

[18] Amer NN Shaaban GM. Association of serum cholesterol ester transfer protein levels with taq IB polymorphism in acute coronary syndrome. Lab Med. (2019) 51(2):199–210. 10.1093/labmed/lmz043 31504738

[19] Iwanicka J Iwanicki T Niemiec P Balcerzyk A Krauze J Górczyńska-Kosiorz S Relationship between CETP gene polymorphisms with coronary artery disease in polish population. Mol Biol Rep. (2018) 45(6):1929–35. 10.1007/s11033-018-4342-1 30178218 PMC6267260

[20] Cai G Shi G Huang Z. Gender specific effect of CETP rs708272 polymorphism on lipid and atherogenic index of plasma levels but not on the risk of coronary artery disease. Medicine (Baltimore). (2018) 97(49):e13514. 10.1097/MD.0000000000013514 30544452 PMC6310534

[21] Maksoud A El-Garf SM Ali WT Shaaban OS Amer GM N N. Association of cholesterol ester transfer protein taq IB polymorphism with acute coronary syndrome in Egyptian national patients. Lab Med. (2017) 48(2):154–65. 10.1093/labmed/lmw071 28387842

[22] Cyrus C Vatte C Al-Nafie A Chathoth S Al-Ali R Al-Shehri A The impact of common polymorphisms in CETP and ABCA1 genes with the risk of coronary artery disease in Saudi arabians. Hum Genomics. (2016) 10(1):8. 10.1186/s40246-016-0065-3 26936456 PMC4776394

[23] Kaman D İlhan N Akbulut M. TaqIB and severity of coronary artery disease in the Turkish population: a pilot study. Biomol Biomed. (2015) 15(1):9–13. 10.17305/bjbms.2015.157 PMC4365681 25725138

[24] Mehlig K Strandhagen E Svensson P-A Rosengren A Torén K Thelle DS CETP TaqIB genotype modifies the association between alcohol and coronary heart disease: the INTERGENE case-control study. Alcohol. (2014) 48(7):695–700. 10.1016/j.alcohol.2014.08.011 25288221

[25] Abd El-Aziz TA Mohamed RH Hagrass HA. Increased risk of premature coronary artery disease in Egyptians with ABCA1 (R219K), CETP (TaqIB), and LCAT (4886C/T) genes polymorphism. J Clin Lipidol. (2014) 8(4):381–9. 10.1016/j.jacl.2014.06.001 25110219

[26] Wang J Wang LJ Zhong Y Gu P Shao JQ Jiang SS CETP gene polymorphisms and risk of coronary atherosclerosis in a Chinese population. Lipids Health Dis. (2013) 12(1):176. 10.1186/1476-511X-12-176 24283500 PMC4220746

[27] Lu Y Tayebi N Li H Saha N Yang H Heng CK. Association of CETP Taq1B and −629C>A polymorphisms with coronary artery disease and lipid levels in the multi-ethnic Singaporean population. Lipids Health Dis. (2013) 12:85. 10.1186/1476-511X-12-85 23758630 PMC3699414

[28] Rahimi Z Nourozi-Rad R Vaisi-Raygani A Saidi M-R Rahimi Z Ahmadi R Association between cholesteryl ester transfer protein TaqIB variants and risk of coronary artery disease and diabetes mellitus in the population of western Iran. Genet Test Mol Biomarkers. (2011) 15(11):813–9. 10.1089/gtmb.2011.0037 21689002

[29] Kolovou G Vasiliadis I Kolovou V Karakosta A Mavrogeni S Papadopoulou E The role of common variants of the cholesteryl ester transfer protein gene in left main coronary artery disease. Lipids Health Dis. (2011) 10:156. 10.1186/1476-511X-10-156 21899732 PMC3175181

[30] Corella D Carrasco P Amiano P Arriola L Chirlaque MD Huerta JM Common cholesteryl ester transfer protein gene variation related to high-density lipoprotein cholesterol is not associated with decreased coronary heart disease risk after a 10-year follow-up in a Mediterranean cohort: modulation by alcohol consumption. Atherosclerosis. (2010) 211(2):531–8. 10.1016/j.atherosclerosis.2010.03.026 20398902

[31] Poduri A Khullar M Bahl A Sharma YP Talwar KK. A combination of proatherogenic single-nucleotide polymorphisms is associated with increased risk of coronary artery disease and myocardial infarction in Asian Indians. DNA Cell Biol. (2009) 28(9):451–60. 10.1089/dna.2009.0887 19558216

[32] Padmaja N Kumar RM Balachander J Adithan C. Cholesteryl ester transfer protein TaqIB, −629C>A and I405V polymorphisms and risk of coronary heart disease in an Indian population. Clin Chim Acta. (2009) 402(1–2):139–45. 10.1016/j.cca.2008.12.041 19168039

[33] Kaestner S Patsouras N Spathas DH Flordellis CS Manolis AS. Lack of association between the cholesteryl ester transfer protein gene—TaqIB polymorphism and coronary restenosis following percutaneous transluminal coronary angioplasty and stenting: a pilot study. Angiology. (2009) 61(4):338–43. 10.1177/0003319709348297 19815603

[34] Rejeb J Omezzine A Rebhi L Naffeti I Kchok K Belkahla R Association of the cholesteryl ester transfer protein Taq1 B2B2 genotype with higher high-density lipoprotein cholesterol concentrations and lower risk of coronary artery disease in a Tunisian population. Arch Cardiovasc Dis. (2008) 101(10):629–36. 10.1016/j.acvd.2008.09.013 19056069

[35] Meiner V Friedlander Y Milo H Sharon N Ben-Avi L Shpitzen S Cholesteryl ester transfer protein (CETP) genetic variation and early onset of non-fatal myocardial infarction. Ann Hum Genet. (2008) 72(Pt 6):732–41. 10.1111/j.1469-1809.2008.00464.x 18637884 PMC2766633

[36] Hsieh MC Tien KJ Chang SJ Lo CS Hsin SC Hsiao JY Cholesteryl ester transfer protein B1B1 genotype as a predictor of coronary artery disease in Taiwanese with type 2 diabetes mellitus. Metab Clin Exp. (2007) 56(6):745–50. 10.1016/j.metabol.2006.12.023 17512305

[37] Dedoussis GV Panagiotakos DB Louizou E Mantoglou I Chrysohoou C Lamnisou K Cholesteryl ester-transfer protein (CETP) polymorphism and the association of acute coronary syndromes by obesity status in Greek subjects: the CARDIO2000-GENE study. Hum Hered. (2007) 63(3–4):155–61. 10.1159/000099827 17310124

[38] Yilmaz H Isbir T Agachan B Karaali ZE. Effects of cholesterol ester transfer protein Taq1B gene polymorphism on serum lipoprotein levels in Turkish coronary artery disease patients. Cell Biochem Funct. (2005) 23(1):23–8. 10.1002/cbf.1124 15386541

[39] Whiting BM Anderson JL Muhlestein JB Horne BD Bair TL Pearson RR Candidate gene susceptibility variants predict intermediate end points but not angiographic coronary artery disease. Am Heart J. (2005) 150(2):243–50. 10.1016/j.ahj.2004.08.034 16086925

[40] Falchi A Giovannoni L Piras IS Calo CM Moral P Vona G Prevalence of genetic risk factors for coronary artery disease in Corsica Island (France). Exp Mol Pathol. (2005) 79(3):210–3. 10.1016/j.yexmp.2005.09.005 16248996

[41] Keavney B Palmer A Parish S Clark S Youngman L Danesh J Lipid-related genes and myocardial infarction in 4685 cases and 3460 controls: discrepancies between genotype, blood lipid concentrations, and coronary disease risk. Int J Epidemiol. (2004) 33(5):1002–13. 10.1093/ije/dyh275 15256516

[42] Andrikopoulos GK Richter DJ Needham EW Zairis MN Karabinos EN Gialafos EJ Association of the ile405val mutation in cholesteryl ester transfer protein gene with risk of acute myocardial infarction. Heart. (2004) 90(11):1336–7. 10.1136/hrt.2003.019067 15486139 PMC1768536

[43] Isbir T Yilmaz H Agachan B Karaali ZE. Cholesterol ester transfer protein, apolipoprotein E and lipoprotein lipase genotypes in patients with coronary artery disease in the Turkish population. Clin Genet. (2003) 64(3):228–34. 10.1034/j.1399-0004.2003.00137.x 12919138

[44] Freeman DJ Samani NJ Wilson V McMahon AD Braund PS Cheng S A polymorphism of the cholesteryl ester transfer protein gene predicts cardiovascular events in non-smokers in the west of Scotland coronary prevention study. Eur Heart J. (2003) 24(20):1833–42. 10.1016/j.ehj.2003.07.001 14563342

[45] Liu S Schmitz C Stampfer MJ Sacks F Hennekens CH Lindpaintner K A prospective study of TaqIB polymorphism in the gene coding for cholesteryl ester transfer protein and risk of myocardial infarction in middle-aged men. Atherosclerosis. (2002) 161(2):469–74. 10.1016/S0021-9150(01)00673-6 11888533

[46] Wu JH Lee YT Hsu HC Hsieh LL. Influence of CETP gene variation on plasma lipid levels and coronary heart disease: a survey in Taiwan. Atherosclerosis. (2001) 159(2):451–8. 10.1016/S0021-9150(01)00524-X 11730826

[47] Arca M Montali A Ombres D Battiloro E Campagna F Ricci G Lack of association of the common TaqIB polymorphism in the cholesteryl ester transfer protein gene with angiographically assessed coronary atherosclerosis. Clin Genet. (2001) 60(5):374–80. 10.1034/j.1399-0004.2001.600510.x 11903340

[48] Bordoni L Samulak JJ Sawicka AK Pelikant-Malecka I Radulska A Lewicki L Trimethylamine N-oxide and the reverse cholesterol transport in cardiovascular disease: a cross-sectional study. Sci Rep. (2020) 10(1):18675. 10.1038/s41598-020-75633-1 33122777 PMC7596051

[49] Arikan GD Isbir S Yilmaz SG Isbir T. Characteristics of coronary artery disease patients who have a polymorphism in the cholesterol ester transfer protein (CETP) gene. In Vivo. (2019) 33(3):787–92. 10.21873/invivo.11540 31028198 PMC6559892

[50] Mirhafez SR Avan A Khatamianfar S Ghasemi F Moohebati M Ebrahimi M There is an association between a genetic polymorphism in the ZNF259 gene involved in lipid metabolism and coronary artery disease. Gene. (2019) 704:80–5. 10.1016/j.gene.2019.02.101 30902787

[51] Wu N Liu G Huang Y Liao Q Han L Ye H Study of the association of 17 lipid-related gene polymorphisms with coronary heart disease. Anatol J Cardiol. (2018) 19(6):360–7. 10.14744/AnatolJCardiol.2018.23682 29848931 PMC5998860

[52] Devi A Singh R Dawar R Tyagi S. Association of cholesteryl ester transfer protein (CETP) gene −629C/A polymorphism with angiographically proven atherosclerosis. Indian J Clin Biochem. (2017) 32(2):235–8. 10.1007/s12291-016-0585-6 28428701 PMC5382070

[53] Goodarzynejad H Boroumand M Behmanesh M Ziaee S Jalali A. Cholesteryl ester transfer protein gene polymorphism (I405V) and premature coronary artery disease in an Iranian population. Bosn J Basic Med Sci. (2016) 16(2):114–20. 10.17305/bjbms.2016.942 26773179 PMC4852992

[54] Ganesan M Nizamuddin S Katkam SK Kumaraswami K Hosad UK Lobo LL C.*84G>A mutation in CETP is associated with coronary artery disease in south Indians. PLoS One. (2016) 11(10):e0164151. 10.1371/journal.pone.0164151 27768712 PMC5074517

[55] Zende PD Bankar MP Momin AR Kamble PS. Study of cholesteryl ester transfer protein (CETP) I405v genotype and its association with lipid fractions in myocardial infarction patients: a case control study. J Clin Diagn Res. (2014) 8(6):Cc01–4. 10.7860/JCDR/2014/7818.4441 25120972 PMC4129287

[56] Xu L Zhou J Huang S Huang Y Le Y Jiang D An association study between genetic polymorphisms related to lipoprotein-associated phospholipase A(2) and coronary heart disease. Exp Ther Med. (2013) 5(3):742–50. 10.3892/etm.2013.911 23404648 PMC3570076

[57] Tanrikulu S Ademoglu E Gurdol F Mutlu-Turkoglu U Bilge AK Nisanci Y. Association of cholesteryl ester transfer protein −629C > A polymorphism with high-density lipoprotein cholesterol levels in coronary artery disease patients. Cell Biochem Funct. (2009) 27(7):452–7. 10.1002/cbf.1593 19784962

[58] Zee RY Cook NR Cheng S Erlich HA Lindpaintner K Ridker PM. Multi-locus candidate gene polymorphisms and risk of myocardial infarction: a population-based, prospective genetic analysis. J Thromb Haemost. (2006) 4(2):341–8. 10.1111/j.1538-7836.2006.01754.x 16420563

[59] Zheng K Zhang S Zhang L He Y Liao L Hou Y Carriers of three polymorphisms of cholesteryl ester transfer protein gene are at increased risk to coronary heart disease in a Chinese population. Int J Cardiol. (2005) 103(3):259–65. 10.1016/j.ijcard.2004.08.065 16098387

[60] Tobin MD Braund PS Burton PR Thompson JR Steeds R Channer K Genotypes and haplotypes predisposing to myocardial infarction: a multilocus case-control study. Eur Heart J. (2004) 25(6):459–67. 10.1016/j.ehj.2003.11.014 15039125

[61] Kontush A. HDL-mediated mechanisms of protection in cardiovascular disease. Cardiovasc Res. (2014) 103(3):341–9. 10.1093/cvr/cvu147 24935434

[62] Lincoff AM Nicholls SJ Riesmeyer JS Barter PJ Brewer HB Fox KAA Evacetrapib and cardiovascular outcomes in high-risk vascular disease. N Engl J Med. (2017) 376(20):1933–42. 10.1056/NEJMoa1609581 28514624

[63] Schwartz GG Olsson AG Abt M Ballantyne CM Barter PJ Brumm J Effects of dalcetrapib in patients with a recent acute coronary syndrome. N Engl J Med. (2012) 367(22):2089–99. 10.1056/NEJMoa1206797 23126252

[64] Cannon CP Shah S Dansky HM Davidson M Brinton EA Gotto AM Safety of anacetrapib in patients with or at high risk for coronary heart disease. N Engl J Med. (2010) 363(25):2406–15. 10.1056/NEJMoa1009744 21082868

[65] Barter PJ Caulfield M Eriksson M Grundy SM Kastelein JJ Komajda M Effects of torcetrapib in patients at high risk for coronary events. N Engl J Med. (2007) 357(21):2109–22. 10.1056/NEJMoa0706628 17984165

[66] The international HapMap project. Nature. (2003) 426(6968):789–96. 10.1038/nature02168 14685227

[67] Manolio TA Collins FS Cox NJ Goldstein DB Hindorff LA Hunter DJ Finding the missing heritability of complex diseases. Nature. (2009) 461(7265):747–53. 10.1038/nature08494 19812666 PMC2831613

